# Female *Aedes aegypti* mosquitoes use communal cues to manage population density at breeding sites

**DOI:** 10.1038/s42003-024-05830-5

**Published:** 2024-01-31

**Authors:** Andre Luis Costa-da-Silva, Silvia Cabal, Kristian Lopez, Jean Boloix, Brian Garcia Rodriguez, Kaylee M. Marrero, Anthony J. Bellantuono, Matthew DeGennaro

**Affiliations:** 1 Department of Biological Sciences, Florida International University, Miami, FL 33199 USA; 2 Biomolecular Sciences Institute, Florida International University, Miami, FL 33199 USA

**Keywords:** Social behaviour, Olfactory receptors

## Abstract

Where a female mosquito lays her eggs creates the conditions for reproductive success. Here we identify a communal behavior among ovipositing female mosquitoes. When choosing equal breeding sites, gravid *Aedes aegypti* aggregate more often than expected. This aggregation occurs when water contact is restricted and does not require the presence of eggs. Instead, the aggregation is regulated by the number of females present at the breeding site. Using assays with both occupied and empty oviposition sites, we show that the Orco olfactory co-receptor and a carbon dioxide receptor, Gr3, detect the presence of mosquitoes. *orco* mutants aggregate more often in empty sites, suggesting attractive olfactory cues influence females to associate with one another. *Gr3* mutant females do not prefer either site, suggesting that the CO_2_ receptor is necessary to evaluate mosquito population density at breeding sites. Further, raising CO_2_ levels is sufficient to cause wild-type mosquitoes to avoid empty oviposition sites. Our results demonstrate that female mosquitoes can regulate their own population density at breeding sites using attractive and repellent communal cues.

## Abstract (web summary)

When choosing equal breeding sites, gravid *Aedes aegypti* aggregate more often than expected. This aggregation depends on the number of females present and involves a density-regulated avoidance response that relies on Orco and the CO2 receptor, Gr3.

## Introduction

Insect species range from living in near-total isolation to forming complex social networks^1^. Mosquitoes are typically characterized as solitary insects^2^. Efforts to describe any interactions among individuals of the same species in mosquitoes have been focused on mating behavior^3^. Male swarms, the aerial aggregation of males to attract females preceding mating, is a phenomenon exhibited by several mosquito species^3^. Laboratory and semi-field experiments have shown that mating swarms enhance mating success by maximizing the encounters between male and female mosquitoes. Male-released pheromones stimulate aggregation of males and attract females to the swarm^4,5^. Interestingly, two studies have shown that *Aedes aegypti* female mosquitoes can attract conspecific females during mating^5,6^. The desire to implement new mosquito control techniques to block disease transmission, many of which involve successful mating events and fecundity, has increased interest in the conspecific aspects of mosquito behavior^7,8^. However, interactions among female mosquitoes have been poorly characterized.

Oviposition behaviors in mosquitoes are likely performed to maximize the success of the progeny^9^. To lay an egg, mosquitoes go through a series of behavioral steps, including site-seeking behavior^9^ and evaluating water quality using sensilla on their legs and proboscis before making the final decision to oviposit^10^. Recently, geosmin has been shown to be an odor cue guiding females to breeding sites in the laboratory and the field^11^. Mosquito eggs and larvae have also been shown to influence egg-laying decisions by providing chemosensory cues to females seeking a breeding site^12–16^, but communication between adult female mosquitoes during the breeding site-seeking behavior has not been previously characterized. Given the importance of *Ae. aegypti* females as the main vectors of arboviruses^17^, conspecific communication of adult mosquitoes may offer a new basis for control methods^4–6,8,18^

In this study, we found that breeding-site seeking *Ae. aegypti* females aggregate more often than expected in one site over others, when equal oviposition site choices are offered. This aggregation occurs regardless of oviposition activity, water contact, or if skip-oviposition is permitted. We determined that the aggregation depends on the number of females present. In addition, a density-regulated avoidance response involving *orco* and *Gr3* pathways govern this behavior. Wild-type females avoid chambers with densely pre-placed females. This response is enhanced in *orco* mutant gravid females that aggregate more often than expected in empty breeding sites, suggesting that attractive olfactory cues influence aggregation. However, *Gr3* mutants do not avoid populated sites. Further, increasing the concentration of carbon dioxide caused females to avoid unoccupied oviposition sites, suggesting that carbon dioxide is necessary and sufficient to produce the avoidance response that modulates the level of aggregation. Altogether, we demonstrate that *Ae. aegypti* mosquitoes can manage population density at breeding sites by using chemosensory-dependent communal cues to orient oviposition choices.

## Results

### Breeding site seeking *Ae. aegypti* females aggregate more often than expected between equal breeding sites

**Fig. 1:**
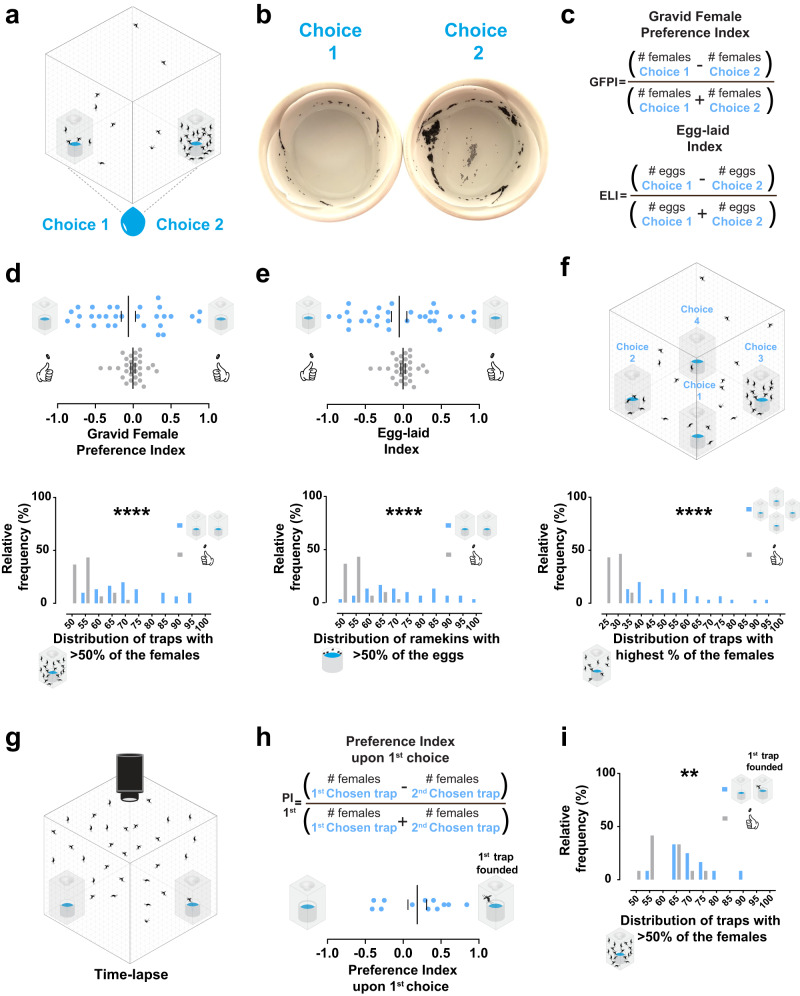
*Ae. aegypti* females randomly aggregate in a breeding site. **a** Illustration showing the two-choice water trap assay with equal breeding choices (Choice 1 and Choice 2) and females aggregated in the trap on the right. Each acrylic trap housed a ramekin lined with filter paper and filled with 10 mL of deionized water. **b** Representative image of an observed aggregation of laid eggs in the assay. **c** Formulas to calculate the gravid female preference index (GFPI) and egg-laid index (ELI). **d**, **e** Results of gravid females trapped (**d**) and eggs laid (**e**) in the assay (*n* = 30 trials, 35 gravid females per trial, 24 h assay). **d top** Preference indices from simulated data (random chances, coin toss icons) or from tested gravid females. No significant skewness of the simulated data (gray dots, *p* = 0.9055) or preference of gravid females toward Choice 1 or Choice 2 (blue dots, *p* = 0.4992) was detected in all trials against the theoretical value (0) by one-sample *t*-test. **d bottom** Expected (gray bars) and observed (blue bars) distributions of traps with aggregated gravid females (>50%) in all trials. The observed median percentage was significantly higher than the simulated data (****p < 0.0001) by the Mann–Whitney test. **e top** Values from simulated data (random chances, coin toss icons) or from counted laid eggs. No significant skewness of the simulated data (gray dots, *p* = 0.9055) or egg-laying preference in Choice 1 or Choice 2 (blue dots, *p* = 0.4992) was detected in all trials against the theoretical value (0) by One-sample *t*-test. **e bottom** Expected (gray bars) and observed (blue bars) distributions of ramekins with aggregated eggs (>50%) in all trials. The observed median percentage was significantly higher than the simulated data (****p < 0.0001) by the Mann–Whitney test. **f top** Illustration of the four-choice water trap assay with equal acrylic traps (Choice 1, Choice 2, Choice 3, and Choice 4), each housing a porcelain ramekin lined with filter paper and filled with 10 mL of deionized water. **f bottom** Expected (25%, gray bars) and observed (blue bars) distributions of traps with aggregated gravid females (highest observed %) in all 4-choice trials (*n* = 30 trials, 35 gravid females per trial, 24 h assay). The observed median percentage was significantly higher than the simulated data (****p < 0.0001) by the Mann–Whitney test. **g** Illustration of a time-lapsed two-choice water trap assay with equal choices. The first trap chosen by a female is depicted (on the right). **h top** Formula to calculate the gravid female preference index upon the first choice (PI 1st). **h bottom and i** Results of the time-lapsed assays (*n* = 12 trials, 35 gravid females per trial, 24 h assay). **h bottom** Gravid females did not have a significant preference for the first trap found (*p* = 0.1573, against theoretical value 0) by One-sample *t*-test. **i** Expected (gray bars) and observed (blue bars) distributions of traps with aggregated gravid females (>50%) in all trials. The observed mean percentage was significantly higher than the simulated data (**p = 0.0096) by the Unpaired *t*-test. A blue dot represents one observed trial, a gray dot represents one simulated trial, a long vertical black line denotes the mean, and a black vertical short line represents the standard error of the mean.

Choice assays have been previously conducted with *Ae. aegypti* female mosquitoes to assess their preference for oviposition sites with different stimuli, and a number of extrinsic factors that influence a female’s decision to lay her eggs have been revealed^10,11,14,19–21^. To test whether oviposition site selection is influenced by intrinsic factors from the female mosquitoes themselves, we used a two-choice water trap assay^21^ for oviposition (Fig. 1a). Over 24 h, gravid *Ae. aegypti* females could choose between two equal breeding sites containing water-filled ramekins housed in transparent traps (Fig. 1a). If females choose randomly between two equal breeding sites, no preference for either trap or egg-laying preference should be expected. However, an aggregation of gravid females inside one trap (represented in Fig. 1a, trap on the right) and clustered eggs laid in the corresponding ramekin (Fig. 1b, ramekin on the right) was often observed.

To confirm that a preference never manifests to any equal choice over multiple trials, we calculated the gravid female preference index (Fig. 1c, top) using the number of females trapped in each choice per assay and the egg-laid index (Fig. 1c, bottom) by counting the number of eggs laid in each ramekin. As expected, gravid females did not show any significant preference between equal choices (blue dots, Fig. 1d top) when the mean of all trials was compared to zero (no preference). Similarly, no egg-laid preference was observed (blue dots, Fig. 1e top). However, the distribution of the preference values per trial (blue dots, Fig. 1d, e, top) led us to evaluate whether a random aggregation occurs more often than expected.

We defined aggregation as a significantly higher trapping rate than expected in traps with more than half of the trapped females. To obtain the expected frequency, we first simulated values for preference assuming 50% random chance, considering the same number of choice events and replicates for the simulation (coin toss icon, Fig. 1d, e, top). No significant skewness was detected when a preference index was estimated with simulated values (coin toss icon), and the distribution by chance was narrow around zero as expected (gray dots, Fig. 1d, e, top). To test the observed results for aggregation, we then compared the frequency of traps with the highest trapping rates in the assays to the ones from the simulated data. We found that the occurrence of these traps is significantly higher than expected (Fig. 1d bottom). A similar result was observed for the eggs laid (Fig. 1e bottom), supported by the strong and significant correlation between the number of females trapped and eggs laid in the same trap (Supplementary Fig. 1a and Supplementary Fig. 1b). Our results show that the intriguing incidence of aggregation of females and eggs laid between two equal breeding sites cannot be simply explained by random choices of the gravid females.

We asked whether the aggregation persists when more breeding sites are presented to the same number of gravid females. We added two more traps to the water trap assay, and water-filled ramekins were placed inside the four identical traps (Fig. 1f top). We again found that the occurrence of traps showing aggregation is significantly higher than expected (Fig. 1f bottom) when the simulated dataset was set to 25% random chance. We also did not observe significantly different choice percentages due to trap position for the gravid females (Supplementary Fig. 1d) or eggs laid (Supplementary Fig. 1e) among the four choices in all trials. As seen in the two-choice water trap assays (Supplementary Fig. 1a), a strong and significant correlation between the percentage of gravid females trapped and eggs laid was detected (Supplementary Fig. 1f). Taken together, these results show that the aggregation of gravid females in the two- or four-choice water trap assays cannot be explained by stochastic mosquito choices, and this behavior remains robust when more breeding sites are available.

We next aimed to understand whether a founder effect could elicit a preference that could be linked to aggregation. We asked whether the trap chosen by the first female to make a choice would become the preferred one by females. Using time-lapse recordings in the two-choice water trap assays to track the first chosen trap (Fig. 1g) and the preference index upon 1st choice (Fig. 1h top), we have not detected a significant preference of the gravid females towards the first chosen trap (Fig. 1h bottom). However, the occurrence of aggregation was again significant (Fig. 1i). This result suggests that a simple founder effect is not the cause of the aggregation; rather, the aggregation occurs from the interaction of multiple females over time.

### Aggregation occurs regardless of the presence of eggs, water contact, or ability to skip-oviposit

**Fig. 2:**
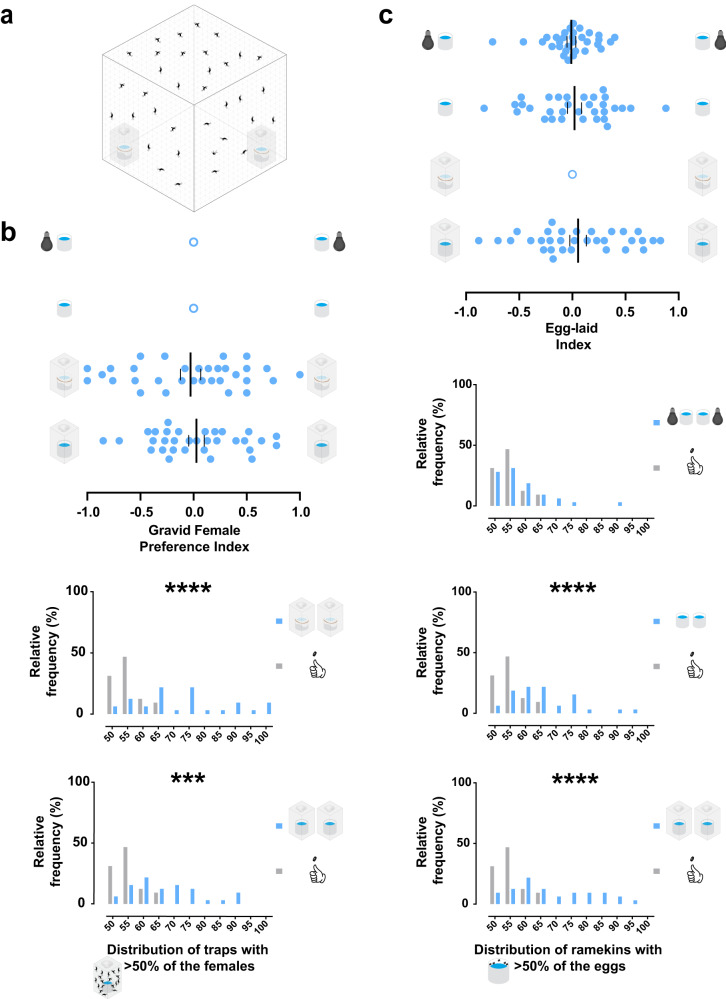
Aggregation is not influenced by laid eggs or skip-oviposition but is reduced in low light. **a** Illustration showing the two-choice water trap assay with equal water-filled ramekins that were obstructed with mesh. **b**, **c** Results of gravid females trapped (**b**) and eggs laid (**c**) in the two-choice assay (*n* = 32 trials, 35 gravid females per trial, 24 h assay). **b top** No significant preference of the gravid females to either equal choice under any condition was detected against the theoretical value (0) by One-sample *t*-test (rows, bottom to top): traps with water-filled ramekins (first row, *p* = 0.7133), traps with water-filled mesh-covered ramekins (second row, *p* = 0.7714). No trapping occurs for the conditions of no traps and water-filled ramekins (third row) and no traps and water-filled ramekins under low light conditions (fourth row). **b bottom** Expected (gray bars) and observed (blue bars) distributions of traps with aggregated gravid females (>50%) in all trials. The mean percentage was significantly higher than the simulated data for traps with water-filled mesh-covered ramekins (top, ****p < 0.0001) and traps with water-filled ramekins (bottom, ***p = 0.0001) by Ordinary one-way ANOVA followed by Dunnett’s multiple comparisons test. **c top** No significant egg-laying preference (blue dots) to either equal choice under any condition was detected against the theoretical value (0) by Wilcoxon signed-rank test (rows, bottom to top): traps with water-filled ramekins (first row, *p* = 0.5452), no traps and water-filled ramekins (third row, *p* = 0.6214), and no traps and water-filled ramekins under low light conditions (fourth row, 0.9884) by Wilcoxon signed-rank test. No egg-laying occurs for the condition traps with water-filled mesh-covered ramekins (second row). **c bottom** Expected (gray bars) and observed (blue bars) distributions of ramekins with aggregated eggs (>50%) in all trials. The observed median percentage of traps and water-filled ramekins under low light conditions was not significantly different (top, *p* > 0.9999), but no traps and water-filled ramekins (middle, ****p < 0.0001) and traps with water-filled ramekins (bottom, ****p < 0.0001) was significantly higher than the simulated data by Kruskal–Wallis test followed by Dunn’s multiple comparison test. A blue dot represents one observed trial, an outlined blue dot represents no data, a long vertical black line denotes the mean, and a black vertical line represents the standard error of the mean.

It has been demonstrated that the presence of eggs influences the oviposition decision of female mosquitoes^18,22^, and the deposition of eggs can shape bacterial communities in the water^23^. We asked whether blocking the ability of the females to contact water or lay eggs in the oviposition site could lead to changes in the aggregation pattern. To answer this question, we returned to our two-choice water trap assay (Fig. 1a), but we covered the ramekins inside the traps with mesh fabric (Fig. 2a). Preventing the gravid females from touching the water to lay eggs did not result in a preference shift towards either choice, as observed for uncovered water-filled ramekins (Fig. 2b), as seen before (Fig. 1d). Gravid females did not have a preference to lay in either site for the uncovered condition (Fig. 2c, with traps), and the egg-laid index cannot be assessed when egg-laying was blocked (Fig. 2c top). When we tested for aggregation, significantly more traps with aggregated females were found for both conditions (Fig. 2b bottom) and were also observed for the eggs laid (Fig. 2c bottom, with traps). We detected that significantly fewer females were trapped when the ramekins were covered with mesh (Supplementary Fig. 2a). Our assay cannot discriminate if this reduction was related to females signaling that the oviposition site is unsuitable, the inability of females to lay eggs or both possibilities. However, a significantly higher than expected aggregation of females was still detected when comparing adjusted simulated data for the mean percentage of females trapped (Supplementary Fig. 2c). Taken together, these results indicate that the aggregation behavior does not require direct contact with water or the presence of eggs.

*Ae. aegypti* gravid females exhibit skip-oviposition, defined as the deposition of eggs by a female in more than one site if two or more options are available^24–30^. To test if the aggregation was simply due to the inability of confined females to skip-oviposit to the other trap, we also performed the assay with the traps removed (Fig. 2c top). When gravid females were free to skip-oviposit between two ramekins, no significant egg-laying preference was observed (Fig. 2c top). However, a significant aggregation of eggs was found in these trials (Fig. 2c bottom, no traps). This suggests that the observed aggregation is not the result of blocking skip-oviposition.

### Egg aggregation ceases in low light but not when visual cues from inside the traps are reduced

Previous studies have shown that *Ae. aegypti* and other mosquito species use visual cues for flight orientation and breeding site location^9,31–33^. We tested whether the aggregation could be disturbed in the dark. When the two-choice assay without traps was performed in low light, no significant egg-laying preference was detected (Fig. 2c top) nor higher egg aggregation than expected (Fig. 2c bottom, light bulbs off). This indicates that visual cues play a role in the aggregation of eggs.

Instead of reducing the visual information from the entire assay, which limits the use of the traps, we investigated whether the visual stimuli from inside the traps could also modulate the occurrence of egg aggregation. We performed two-choice water trap assays with transparent or opaque traps (Supplementary Fig. 2d top). Even though no significant egg-laying preference was detected (Supplementary Fig. 2d top), significant aggregation of eggs was observed for both conditions (Supplementary Fig. 2d bottom). We detected that significantly fewer females were trapped with opaque traps (Supplementary Fig. 2e). However, significant egg aggregation was again found against the adjusted simulated data (Supplementary Fig. 2g). Our findings showed that the aggregation of eggs laid ceases in low light, but reduced visual information from the breeding site was not sufficient to disrupt egg aggregation. Taken together, these results suggest that a broad reduction in visual information can modify their choice decisions, disrupting aggregation. However, the reduction of visual cues when approaching the breeding sites is not as relevant, implying that other sensory modalities can be involved in the aggregation behavior.

### *Ae. aegypti* gravid females also exhibit aggregation in the oviseekmeter

**Fig. 3:**
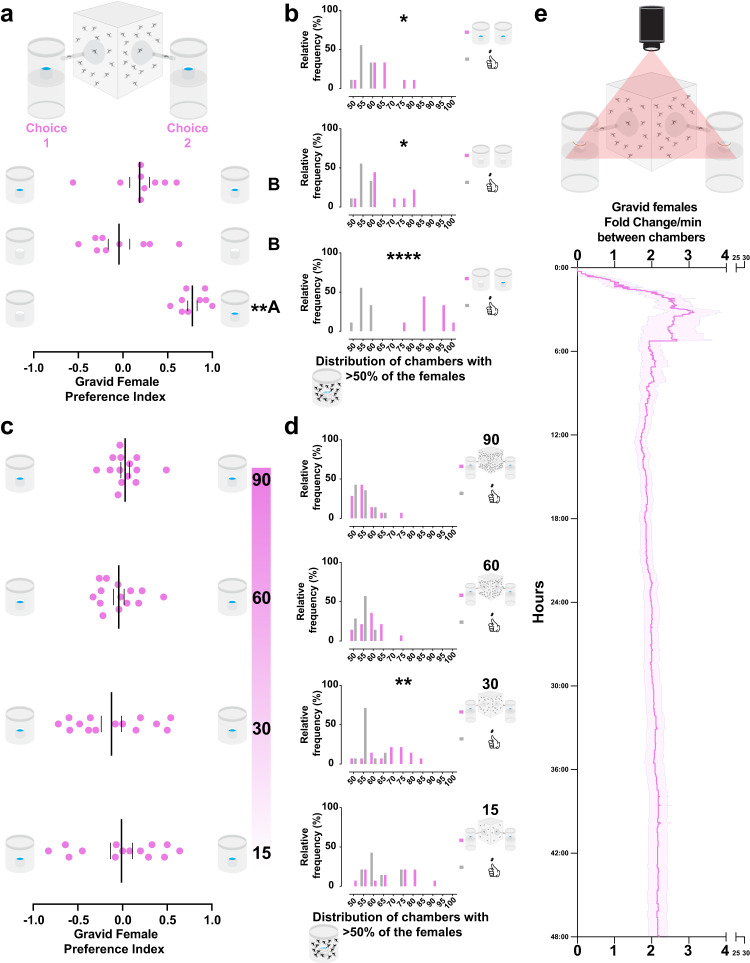
Aggregation is density-dependent and arises from an oscillatory pattern of density-regulated choices. **a**, **b** Oviseekmeter assay and validation. **a top** Illustration of the oviseekmeter, a cage connected to two chambers by glass funnels. Choice 1 and Choice 2 have equal water-filled ramekins as breeding sites. **a bottom and b** Results of gravid females trapped in the chambers (*n* = 9 trials, 30 mosquitoes per trial, 48 h assay). **a bottom** Gravid females preferred the chamber with a water-filled ramekin over the one with an empty ramekin (first row, **p = 0.0039), but no preference was detected when both chambers had empty ramekins (second row, *p* = 0.6797) or both chambers had water-filled ramekins (third row, *p* = 0.1289) (tested against the theoretical value 0) by Wilcoxon signed-rank test. The results with equal ramekins are not significantly different from each other (*p* > 0.9999) but are significantly different from the results with only a ramekin with water (***p = 0.0002, water-filled ramekins and **p = 0.0060, empty ramekins), by Kruskal–Wallis test followed by Dunn’s multiple comparisons test. **b** Expected (gray bars) or observed (pink bars) distributions of chambers with aggregated gravid females (>50%) in all trials. The observed mean percentage was significantly higher than the simulated data for both chambers with water-filled ramekins (top, *p = 0.0224), both chambers with empty ramekins (middle, *p = 0.0375), and only one chamber with water-filled ramekin (bottom, ****p < 0.0001) by Ordinary one-way ANOVA followed by Dunnett’s multiple comparisons test. **c**, **d** Oviseekmeter results with 15, 30, 60, and 90 gravid females released when both chambers had equal water-filled ramekins (*n* = 14 trials, 48 h assay). No preference for either equal choice when 15 (first row, *p* = 0.9279), 30 (second row, 0.2948), 60 (third row, 0.4964), or 90 (fourth row, *p* = 0.5486) gravid females were released was detected (against the theoretical value 0) by One-sample *t*-test. No significant difference was detected among groups by Ordinary one-way ANOVA (*p* = 0.6858). **d** Expected (gray bars) or observed (pink bars) distributions of chambers with aggregated gravid females (>50%) in all trials. The observed median percentage was significantly higher than the simulated data for 30 (**p = 0.0034) but not for 15 (*p* > 0.9999), 60 (*p* = 0.4487), and 90 (*p* > 0.9999) by the Kruskal–Wallis test followed by Dunn’s multiple comparisons test. **e top** Illustration of a time-lapsed oviseekmeter assay. The red triangle represents the imaging field between the two chambers. **e bottom** Scored choice results of the gravid females over 48 h from time-lapse videos (*n* = 9 trials, 30 mosquitoes per trial). The pink line indicates the mean level of fold change per minute between both chambers and the pink-shaded area with purple outline indicates the standard error of the mean. A pink dot represents one observed trial, a long vertical black line denotes the mean, and a black vertical short line represents the standard error of the mean. Different letters mark whether a group of trials is significantly different.

We designed a two-choice oviposition behavior assay, the oviseekmeter (Fig. 3a top), to test whether the aggregation is consistent regardless of the testing device and to allow us to study this behavior with larger numbers of females without overcrowding the breeding site. The oviseekmeter has two choice chambers hosting breeding sites. The choice chamber (Fig. 3a top) is three times larger in volume (~465 cm^3^) than the trap used in the two-choice water trap assay (~144 cm^3^) (Fig. 1a). Each sealed chamber is connected to the main cage by a funnel-shaped narrow passage. This creates independent microenvironments isolated from the main cage from where the gravid females are released (Fig. 3a top). To test whether the mosquitoes can discriminate a choice in the oviseekmeter (Fig. 3a top), gravid females were released for 48 h in an oviseekmeter where an empty ramekin was placed in one chamber and a water-filled ramekin in the other. The females showed a significant preference toward the chamber with the ramekin containing water (Fig. 3a bottom). However, no significant preference was observed when both chambers had empty ramekins or water-filled ramekins (Fig. 3a bottom). The preference levels from trials with equal choices also differed significantly from the ones with only one chamber with a water-filled ramekin (Fig. 3a bottom). To validate whether the random aggregation behavior occurs in this device, we tested the chambers for aggregation, comparing the trapping results to the simulated data. We detected female aggregation regardless of the condition (Fig. 3b). However, the aggregation was directional only when one water-filled ramekin was present (Fig. 3a, b, bottom). We showed that the gravid females can discriminate between choices and aggregate in the oviseekmeter, confirming its suitability to study the aggregation behavior. We also found that the aggregation of gravid females occurred regardless of a water source in the breeding site, since a mean trapping percentage of 85% was observed when the ramekins were empty (Supplementary Fig. 3a). Our data indicates that gravid females are actively seeking a breeding site instead of making choices driven by thirst. In trials with sugar-fed females in the oviseekmeter, a much lower mean trapping percentage, around 30%, was observed when ramekins were empty (Supplementary Fig. 3c).

### Aggregation depends on population density and is established through an oscillatory pattern of density-regulated choices

We asked whether the aggregation behavior remains similar regardless of the number of females in interaction to seek a breeding site. We used the oviseekmeter with water-filled ramekins in the chambers (Fig. 3a top) and released a range of gravid females—15, 30, 60, and 90; no side bias was observed for either chamber (Fig. 3c). Surprisingly, aggregation was only detected when 30 females were tested in contrast to assays with greater or fewer mosquitoes (Fig. 3d). Additionally, we found that there is a strong and significant negative linear association between the observed number of chambers with more than half of the females and population density (Supplementary Fig. 3d). No significant association was detected for the simulated data (Supplementary Fig. 3d). These results suggest that the aggregation of the gravid females is density-dependent and indicate that the females no longer aggregate when there are too few or too many mosquitoes.

To characterize the dynamic formation of the aggregation through a time-course of the choices in the oviseekmeter, we used a time-lapse camera pointed to both entrance regions of the two chambers (Fig. 3e). In 9 trials, we time-lapsed the choices of 30 gravid females released in the oviseekmeter over the course of 48 h, and we scored the choices by watching the videos. The video scoring was consistent with the counted numbers when the experiment was concluded (Supplementary Fig. 3g). The mean fold change per minute between chambers showed a dynamic of choices in which most of the gravid females chose a chamber in the first 24 h. The time course revealed an oscillating pattern of choices between the two chambers (Fig. 3e bottom). From 0 to 24 h, the leading chamber was counterbalanced multiple times by choices in the opposite chamber until the aggregation was established (Fig. 3e bottom and Supplementary Fig. 3i). After 24 h, the fold change between chambers remained more stable until the assay was completed (Fig. 3e bottom), likely due to fewer females left to make a choice. Remarkably, we observed that the mean final fold change was 2.14, but the mean fold change over the assay never surpassed 4-fold (Fig. 3e bottom). We confirmed that the occurrence of traps with aggregation was significant after 48 h, showing that this behavior also occurred in these assays (Supplementary Fig. 3h). Moreover, we noticed that the chamber with a higher percentage of females trapped was not always the first to be chosen (Supplementary Fig. 3i, trials 3 and 4), as seen previously (Fig. 1h bottom). Taken together, our findings suggest that aggregation arises from an oscillatory pattern that is regulated by a density-dependent choice mechanism that has a tolerance threshold (fold change), consistent with the observed occurrence of aggregation in the assays in this study.

### Densely pre-occupied chambers are avoided by breeding site-seeking females, a response that enhances aggregation

**Fig. 4:**
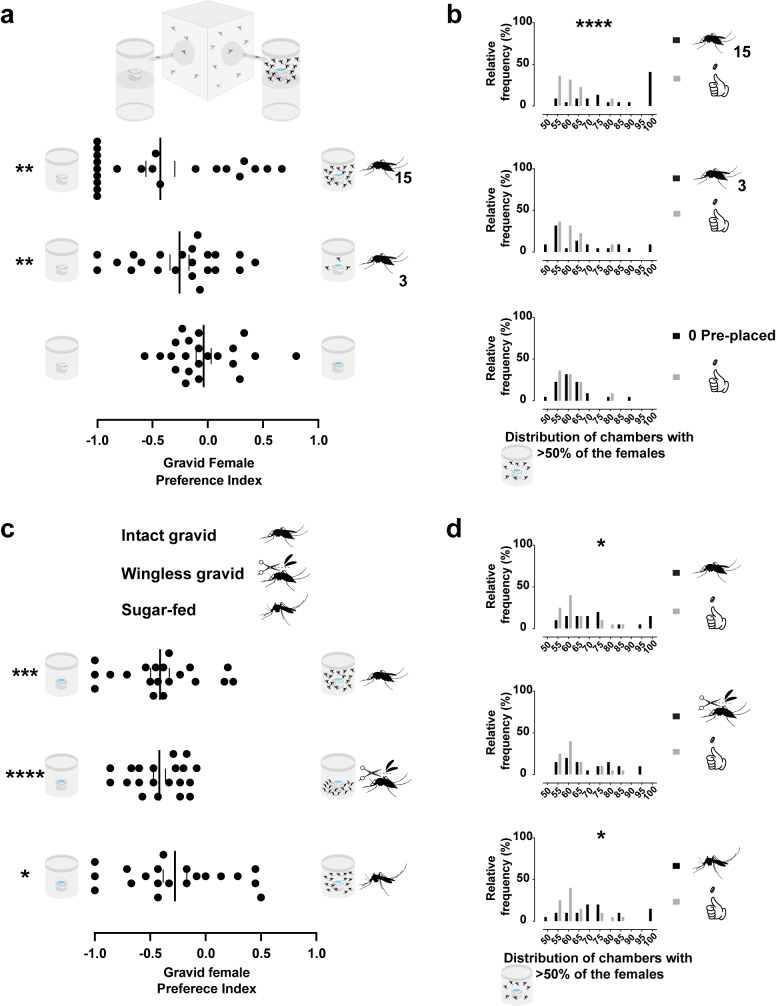
Gravid females avoid pre-occupied chambers, a response that enhances aggregation. **a top** Schematic representation of the oviseekmeter with 15 pre-placed gravid females in only one chamber, both containing meshed water-filled ramekins. **a bottom** and **b** Results of gravid females when 0, 3, or 15 females were pre-placed in one of the chambers (*n* = 22 trials, 15 mosquitoes per trial, 48 h assay). **a bottom** Gravid females preferred unoccupied chambers over chambers with 3 (**p = 0.0086) or 15 (**p = 0.0040) pre-placed gravid females, but no significant preference was detected for assays without pre-placed gravid females (*p* = 0.5327) (tested against the theoretical value 0) by Wilcoxon signed-rank test. No significant difference was detected among groups by the Kruskal–Wallis test followed by Dunn’s multiple comparison test (*p* = 0.0589). **b** Expected (gray bars) or observed (pink bars) distributions of chambers with aggregated gravid females (>50%) in all trials. The observed median percentage was significantly higher than the simulated data when 15 gravid females were pre-placed (****p < 0.0001), but not when 3 (*p* = 0.4975) or none (*p* > 0.9999) by Kruskal–Wallis test followed by Dunn’s multiple comparisons test. **c top** Schematic representation of intact gravid, wingless gravid, and sugar-fed females. **c bottom and d** Results of gravid females released in the oviseekmeter when 15 females for each condition were pre-placed in one of the chambers, with both containing equal meshed water-filled ramekins (*n* = 20 trials, 15 mosquitoes per trial, 48 h assay). **c bottom** Gravid females preferred unoccupied chambers over chambers with 15 sugar-fed (*p = 0.0186), wingless gravid (****p < 0.0001), or intact gravid females (***p = 0.001) (against the theoretical value 0) by One-sample *t*-test. No significant difference was detected among groups (0.8812) by Ordinary one-way ANOVA. **d** Expected (gray bars) or observed (black bars) distributions of chambers with aggregated gravid females (>50%) in all trials. The observed median percentage was significantly higher than the simulated data when 15 sugar-fed (*p = 0.0441) or intact gravid females (*p = 0.0238) were pre-placed, but not for wingless gravid (*p* = 0.0868) by Kruskal–Wallis test followed by Dunn’s multiple comparisons test. A black dot represents one observed trial, a long vertical black line denotes the mean, and a black vertical short line represents the standard error of the mean.

Finding a relationship between mosquito aggregation and population density, we asked whether the occupation status of a chamber could modulate the choice of breeding-site-seeking females. We decided to pre-place 3 and 15 gravid females in only one of the two chambers to observe the choice behavior of 15 gravid females released (Fig. 4a top, 15 pre-placed females depicted). Mesh-covered water-filled ramekins were used in each chamber to prevent oviposition activity of the pre-placed females (Fig. 4a top). Without pre-placed gravid females, no significant preference was found for either chamber (Fig. 4a bottom). However, significant preferences were detected towards the empty chambers when 15 (mean −0.42; *p* = 0.0040, Wilcoxon signed-rank test) or 3 (mean −0.22; *p* = 0.0086, Wilcoxon signed-rank test) gravid females were pre-placed. We did not detect a significant difference among groups in a multiple comparison analysis (Fig. 4a bottom, Supplementary Fig. 4a and Supplementary Fig. 4b). Surprisingly, however, we found that the occurrence of aggregation was significant when only 15 gravid females were pre-placed, but not for 3 pre-placed females or none (Fig. 4b). This suggests that the moderate avoidance with 15 pre-placed females was sufficient to cause a shift in the aggregation pattern (Fig. 4a bottom and 4b).

We further explored this avoidance response producing directional aggregation by performing oviseekmeter assays with pre-placed wingless gravid, intact gravid, or sugar-fed females (Fig. 4c top). Using wingless females allowed us to ensure the pre-trapped ones did not escape from the chambers. We also performed assays with pre-placed sugar-fed females in one of the chambers to test whether the physiological state would change the avoidance response. As expected, we found that the gravid females seeking a breeding site significantly avoided (mean −0.41; *p* = 0.001, One-sample *t*-test) the chambers with pre-placed intact gravid females (Fig. 4c bottom), consistent with previous results (Fig. 4a bottom). The gravid females also avoided the chambers when wingless (mean −0.41; *p* < 0.0001, One-sample *t*-test) or sugar-fed females (mean −0.27; *p* = 0.0186, One-sample *t*-test) were pre-placed, and no significant difference was found among the groups (Fig. 4c bottom, Supplementary Fig. 4c and Supplementary Fig. 4d). When we tested for aggregation, the occurrence of traps with aggregated females was significant when only 15 intact gravid females or sugar-fed females were pre-placed, but not for wingless gravid females (Fig. 4d bottom). Even though this result might suggest that the wingbeat cues modulate the direction of the aggregation, our method is limited to addressing this sensory modality. We also cannot rule out that the winged females can leave the chambers, producing a different interaction. Altogether, the results showed that there is a density-dependent shift that causes more frequent directional aggregation in empty chambers.

### Odorant receptors participate in interactions among gravid females

**Fig. 5:**
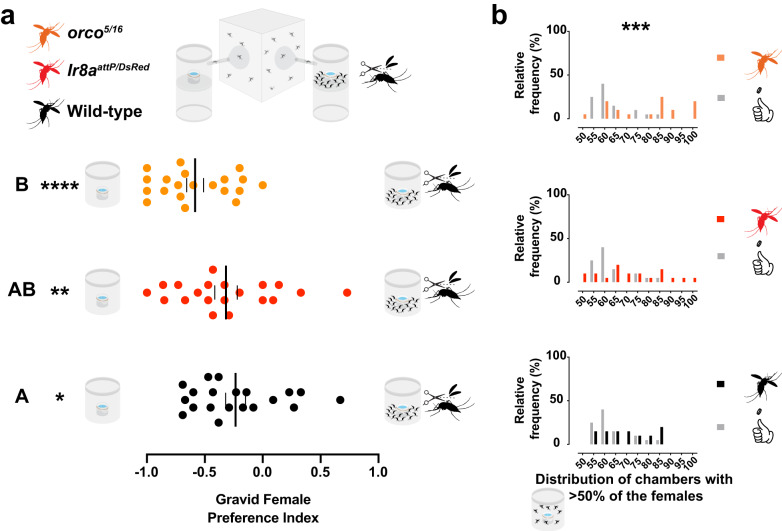
*orco* mutant females show directional aggregation towards the empty chambers. **a top** Schematic representation of Wild-type (black), *Ir8a*^*attP/DsRed*^ (red), and *orco*^*5/16*^ (orange) mutant mosquitoes (left) released in the oviseekmeter (right). **a bottom** and **b** Results of gravid females from the 3 genotypes when 15 wild-type wingless gravid females were pre-placed in one chamber, both containing equal mesh-covered water-filled ramekins (*n* = 20 trials, 15 mosquitoes per trial, 48 h assay). **a bottom** Preference indices of gravid females from the three genotypes. Gravid females from wild-type (black dots, *p = 0.0135), *Ir8a*^*attP/DsRed*^ (red dots, *p = 0.0041), and *orco*^*5/16*^ (orange dots, ****p < 0.0001) avoided occupied chambers (tested against the theoretical value 0) by One-sample *t*-test. *orco*^*5/16*^ group differed significantly from wild-type (*p* = 0.0151) but not from *Ir8a*^*attP/DsRed*^ (*p* = 0.0797). *Ir8a*^*attP/DsRed*^ group did not differ significantly from wild-type (*p* = 0.7754) by Ordinary one-way ANOVA followed by Tukey’s multiple comparisons test. **b** Expected (gray bars) or observed distributions of chambers with aggregated gravid females (>50%) in all trials. The observed mean percentage for *orco*^*5/16*^ (orange bars, ***p < 0.0005) was significantly higher than the simulated data, but not for wild-type (black bars, *p* = 0.2474) or *Ir8a*^*attP/DsRed*^ (red bars, *p = 0.0673) by Ordinary One-way ANOVA followed by Dunnet’s multiple comparisons test. A black, red, or orange dot represents one observed trial, a long vertical black line denotes the mean, and a black vertical short line represents the standard error of the mean. Different letters mark whether a group of trials is significantly different.

We asked whether olfaction could mediate the conspecific interaction, producing an avoidance response that leads to more frequent directional aggregation. To address this question, we tested *Ae. aegypti* null mutants for the Odorant Receptor (ORs) pathway, Orco^34^, and the Ionotropic Receptor (IRs) pathway co-receptor, IR8a^35^. We released 15 gravid females of each genotype, wild-type, heteroallelic *orco*^*5/16*^, and *Ir8a*^*attP/DsRed*^ mutant gravid females in the oviseekmeter (Fig. 5a top left). Fifteen wingless wild-type gravid females were pre-placed in one of the chambers (Fig. 5a top right). We decided to pre-place wingless gravid females to ensure they do not escape and because they cause avoidance but are less likely to cause aggregation (Fig. 4d). Gravid females from the 3 genotypes showed a preference for the unoccupied chambers (Fig. 5a bottom). Surprisingly, *orco*^*5/16*^ mutants had a significantly stronger avoidance (mean −0.58; p = 0.0151, Ordinary one-way ANOVA followed by Tukey’s multiple comparisons test) to the pre-occupied chamber than the wild-type gravid females (mean −0.23) (Fig. 5a bottom). The avoidance response of *Ir8a*^*attP/DsRed*^ mutant gravid females (mean −0.31; *p* = 0.7754, Ordinary one-way ANOVA followed by Tukey’s multiple comparisons test) was not significantly different from wild-type nor from *orco*^*5/16*^ females, limiting our interpretation on the role of *Ir8a*-dependent ionotropic receptors in this behavior. Remarkably, *orco*^*5/16*^ gravid females showed significantly higher frequency of directional aggregation than expected, but this was not observed for wild-type or *Ir8a*^*attP/DsRed*^ (Fig. 5b). This suggests that the Odorant Receptor pathway may sense cues that help mosquitoes navigate toward each other.

We noted a slight but significantly higher percentage of gravid females trapped (Supplementary Fig. 5a) and lower mortality (Supplementary Fig. 5b) for the olfactory receptor mutant mosquitoes. Both results were strong and significantly negatively correlated (Supplementary Fig. 5c), which suggests that when mosquitoes were more drawn to water the mortality was even lower during the assay. We speculate that mutants with reduced olfactory receptor function may be more attracted to water sources, as has been previously seen in *Ir8a* mutants^21^.

### Carbon dioxide acts as a repellent cue during gravid mosquito aggregation

**Fig. 6:**
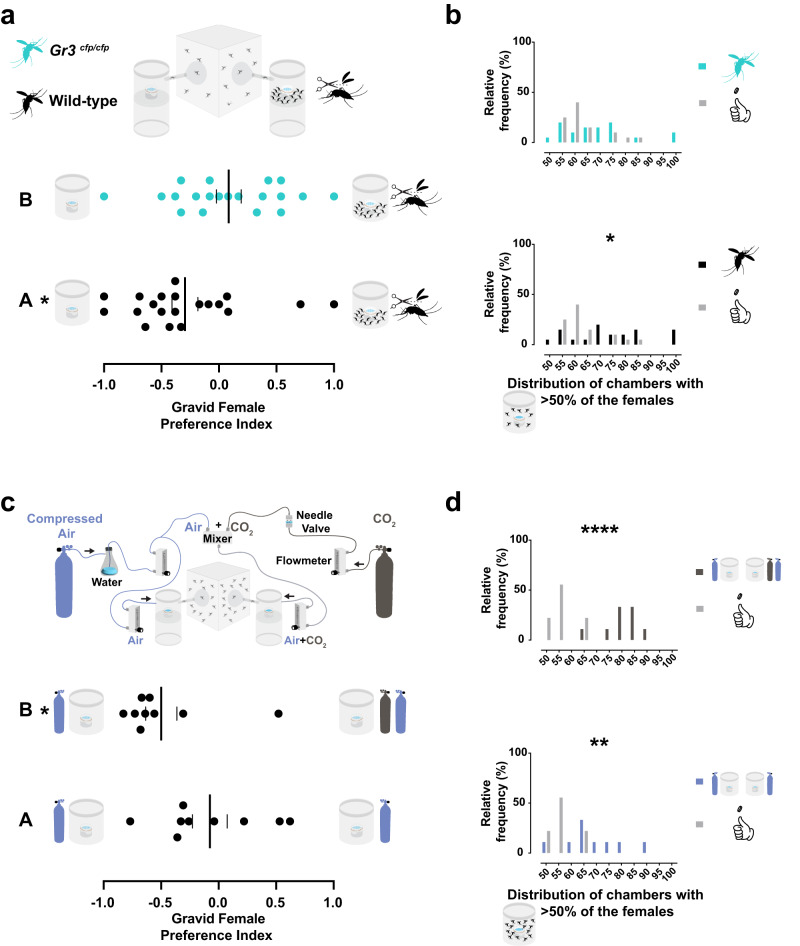
Carbon dioxide is necessary and sufficient for avoidance during gravid mosquito aggregation. **a top** Schematic representation of Wild-type (black), and *Gr3*^*cfp/cfp*^ (cyan) mutant mosquitoes (left) released in the oviseekmeter (right). **a bottom** and **b** Results of gravid females from 2 different genotypes when 15 wild-type wingless gravid females were pre-placed in one chamber, both containing mesh-covered water-filled ramekins (*n* = 20 trials, 15 mosquitoes per trial, 48 h assay). **a bottom** Preference indices of gravid females from the two genotypes. Gravid females from wild-type (black dots, *p = 0.0164) preferred unoccupied chambers, but no significant preference was observed for *Gr3*^*cfp/cfp*^ (cyan dots, *p* = 0.3670) (tested against the theoretical value 0) by One-sample *t*-test. The two groups differed significantly (*p* = 0.0335) by Mann–Whitney test. **b** Expected (gray bars) or observed distributions of chambers with aggregated gravid females (>50%) in all trials. The observed mean percentage for wild-type gravid females (black bars, *p < 0.0205) was significantly higher than the simulated data, but not for *Gr3*^*cfp/cfp*^ (cyan bars, *p* = 0.2253) by Ordinary One-way ANOVA followed by Dunnet’s multiple comparisons test. **c top** Wild-type mosquitoes released in the oviseekmeter coupled with a system to inflow compressed air through the chamber inlet (black arrows indicate the airflow direction). The system supplies  humidified air (dark blue tank and tubing, Erlenmeyer with water and upstream main flowmeter) to a control chamber of the oviseekmeter (bottom left). The system also supplies carbon dioxide (CO_2_) (brown tank and tubing, and upstream main flowmeter) finely regulated by the needle valve and mixed with air when flowing through the mixer. The mixture of air and carbon dioxide (gray tubing) flows to the inlet of the experimental chamber (bottom right). Each chamber had the same flow rate (0.2 L/min) regulated by independent downstream flowmeters. Both chambers of the control oviseekmeter were not supplemented with carbon dioxide (see Methods for details). **c bottom** and **d** Results of wild-type gravid females tested in an oviseekmeter with only one chamber at a higher concentration of carbon dioxide. No females were pre-placed in any chamber, both containing mesh-covered water-filled ramekins (*n* = 9 trials, 30 mosquitoes per trial, 14 h assay). **c bottom** Preference indices from gravid females (black dots) when choosing chambers without supplemented CO_2_ (blue tanks) or only one chamber with supplemented CO_2_ (blue and brown tanks). A significant avoidance was observed against the chamber supplemented with CO_2_ (brown and blue tanks, *p = 0.0117), but no significant preference was observed for either chamber without supplemented CO_2_ (blue tanks, *p* = 0.5703) (tested against the theoretical value 0) by Wilcoxon signed-rank test. Both groups differed significantly (*p* = 0.0071) by the Mann–Whitney test. **d** Expected (gray bars) or observed distributions of chambers with aggregated gravid females (>50%) in all trials. The observed mean percentage was significantly higher than the simulated data when CO_2_ was supplemented into one chamber (brown bars, ****p < 0.0001) or when CO_2_ was not supplemented (blue bars, **p < 0.0205) by Ordinary One-way ANOVA followed by Dunnet’s multiple comparisons test. A black or cyan dot represents one observed trial, a long vertical black line denotes the mean, and a black vertical short line represents the standard error of the mean. Different letters mark whether a group of trials is significantly different.

The results with *orco* mutants suggest that attractive olfactory cues are involved in the directional aggregation behavior but could not explain the avoidance of densely occupied sites. We hypothesized that carbon dioxide (CO_2_) detection could play a role in the avoidance behavior based on studies in *Drosophila*^36–39^. To test our hypothesis, we used *Ae. aegypti Gr3* mutants, which lack a subunit of the heteromeric CO_2_ receptor and do not show electrophysiological or behavioral responses to CO_2_^40^. We released wild-type and homozygous *Gr3*^*cfp/cfp*^ gravid females (Fig. 6a top left) in the oviseekmeter with 15 pre-placed wingless wild-type gravid females in one of the chambers (Fig. 6a top right). Wild-type gravid females significantly avoided the pre-occupied chambers with wingless gravid females (Fig. 6a bottom, mean −0.29; *p* = 0.0164, One-sample *t*-test), consistent with what was observed before (Fig. 4c and Fig. 5a), but the aggregation was detected in these trials (Fig. 6b). This suggests that 15 females may be around the threshold that results in a shift from only preference to significant directional aggregation. Remarkably, the *Gr3*^*cfp/cfp*^ females did not avoid the occupied chambers (Fig. 6a bottom, mean 0.08; *p* = 0.3670, One-sample *t*-test) and the occurrence of traps with aggregated females was not significantly different than expected (Fig. 6b). These results strongly indicate the CO_2_ receptor *Gr3* mediates the avoidance behavior, likely by sensing slight changes in CO_2_ levels altered by the females pre-placed in the chamber. *Gr3*^*cfp/cfp*^ gravid females showed a significantly higher percentage of trapped mosquitoes (Supplementary Fig. 6a) and lower mortality than the wild-type controls (Supplementary Fig. 6b). These results were moderate but significantly negatively correlated (Supplementary Fig. 6c) as previously seen with *orco* and *Ir8a* mutants (Supplementary Fig. 5a and Supplementary Fig. 5b), and may suggest that *Gr3*^*cfp/cfp*^ mutants may also exhibit increased water attraction similar to Ir8a mutants^21^.

Our results indicate that gravid females require the CO_2_ receptor to avoid densely populated chambers containing an oviposition site. We aimed to directly confirm that CO_2_ is the cue mediating the avoidance response against pre-occupied chambers. Using the same behavior assay, we tested whether higher concentrations of CO_2_ would result in an avoidance response like having pre-placed females in the chamber. We used wild-type gravid females in the oviseekmeter with modified chambers that allow airflow via connected tubing (Fig. 6c top). We delivered air inflow without CO_2_ for the control chamber, and with supplemented CO_2_ for the opposite chamber (Fig. 6c top). We were able to produce a concentration difference of 100–250 ppm of CO_2_ in front of the funnel entrances at the main chamber. *Ae. aegypti* wild-type gravid females did not show significant preference for any chambers when CO_2_ was not supplemented (Fig. 6c bottom, mean −0.07; *p* = 0.5703, Wilcoxon signed-rank test). However, the gravid females significantly avoided the chamber supplemented with CO_2_ (Fig. 6c bottom, mean −0.50; *p* = 0.0117, Wilcoxon signed-rank test). Both groups were also significantly different from each other (Fig. 6c bottom). Gravid females showed a significant non-directional aggregation in assays without supplemented CO_2_ (Fig. 6c, d), consistent with assays with 30 females (Fig. 3d). However, a significant directional aggregation was detected when females were in trials with supplemented CO_2_ in a chamber (Fig. 6c, d). Our data also indicates that the increase in CO_2_ concentration was not detrimental to the mosquitoes (Supplementary Fig. 6d and Supplementary Fig. 6e). The avoidance behavior of the released gravid females against the chamber with supplemented CO_2_ mimicked the results with pre-placed females in the chamber (Fig. 4c, Fig. 5a, and Fig. 6a). Collectively, these results strongly suggest that CO_2_ is the avoidance cue that shifts the preference of the gravid females to unoccupied breeding sites, contributing to the formation of directional aggregation.

## Discussion

We demonstrated that female *Ae. aegypti* mosquitoes can use communal information to aggregate or disperse during oviposition-site seeking. Our findings suggest that gravid mosquito females sense and respond behaviorally to the density of individuals in a microenvironment containing breeding sites. The careful regulation of oviposition site occupation by gravid female mosquitoes may be a strategy to maximize mosquito reproductive success. It is likely that the aggregation in lower density can be related to the attested quality of the breeding site by other females. On the contrary, the avoidance response in a higher density can minimize progeny competition. Similar relationships involving the density of eggs and immatures influencing female choice have been demonstrated in laboratory and field studies^15,16,41–47^. Our study established this density-dependent behavior relationship among adult females of *Ae. aegypti*. Recent findings in more solitary insects have revealed that their decision-making processes can also rely on conspecific interactions, leading to shifts in behavioral responses^34,35^. For example, *Drosophila* males rely on olfaction to gauge the number of conspecific individuals in their surroundings and use this density information to alter social distance^48^.

We also showed that distinct olfactory pathways mediate the density-dependent behavioral responses to breeding sites. A recent study found that intraspecific volatile organic compounds (VOCs) from eggs and immature stages can modulate the selection of breeding sites by *Ae. aegypti* females. The quality and quantity of these intraspecific VOCs blends are chemical codes influencing site selection in a density-dependent manner^16^. Our findings demonstrate that intraspecific combined cues from *Ae. aegypti* adult females are likely to function in a similar fashion as a proxy for adult density around the breeding site.

The findings with *Ae. aegypti orco* mutants suggest that there is an olfactory attraction among gravid females that is linked to the OR pathway, indicating two non-mutually exclusive interpretations. The *orco* pathway can inhibit the avoidance or drive an attraction that contributes to aggregation. If *orco* inhibits the avoidance, gravid females would likely choose the chamber with pre-placed females more often. This would result in less frequent aggregation events overall or no significant directional aggregation. However, the strong preference towards the empty chamber and the significant directional aggregation away from pre-placed females (Fig. 5a bottom and Fig. 5b) suggest that the *orco* pathway is likely involved in an attraction response linked to aggregation. Identifying the volatile cues that rely on this pathway, likely intraspecific adult VOCs^16^ would be helpful in determining how the OR pathway allows mosquitoes to navigate toward each other.

Behavioral analysis of *Gr3* mutant mosquitoes allowed us to determine that the avoidance response likely involves carbon dioxide detection. It has been shown that *Gr3* mutants do not sense or show a behavioral response to volatile CO_2_^40^. However, the CO_2_ receptor in mosquitoes can be involved in the detection of other volatiles, such as pyridine^49^. Our CO_2_ avoidance assays strongly suggest that CO_2_ is the main volatile eliciting the avoidance response, even though we cannot rule out other female-emitted volatiles contributing synergistically to the *Gr3*-dependent avoidance response.

Plumes of CO_2_ are crucial for *Ae. aegypti* to become behaviorally active and to engage in host seeking^50,51^, but this activation response is lost in egg-laying females^50,51^. Our results show that higher CO_2_ levels can be a stimulus for an avoidance response and dispersion in gravid females seeking a breeding site, the opposite behavior response found in host-seeking females. CO_2_-mediated avoidance behaviors have also been characterized in *Drosophila* at higher concentrations^36,52^, and yet, in low dosages, CO_2_ may enhance *Drosophila* flight^53,54^.

*Ae. aegy*pti CO_2_ receptor neurons are extremely sensitive to small changes in CO_2_ levels. They respond to step increases in CO_2_ concentration as low as 50 ppm, independently of the background concentration of CO_2_^55^. Due to this ability, it is likely that several females lying in the same site are exhaling more CO_2_ and can increase its concentration in the microenvironment. This can indicate a higher occupation status of the breeding site to following females approaching the site. We also speculate that breeding-site-seeking females may detect the increased concentration of CO_2_ caused by the respiratory activity of immatures in overcrowded breeding sites as a signal for dispersion. It has been shown that the respiration of individual mosquitoes can change the air microenvironment, although the technical approach to measure a group of females can be challenging^56^. For example, movement, physiological state, and flight activity can make the oxygen uptake fluctuate^57^, and it is plausible that females can detect these sudden variations in CO_2_ concentration. This avoidance response triggered by CO_2_ may also be related to defense against predators or human hosts when mosquito females are seeking a breeding site.

It is noteworthy that *orco* mutant gravid females showed strong avoidance of occupied breeding sites, with directional aggregation to the empty sites. This indicates that volatiles detected by ORs produce an opposite and competing attraction behavior. Based on these findings, we speculate that there is a balance between the levels of attractive cues and repellent CO_2_ emitted from conspecific adult females. It is likely that variations in this balance can result in density-dependent shifts in mosquito breeding site selection. Similar examples of opposing behaviors involving two different olfactory sensory pathways have been shown in *Drosophila*, although the behavioral responses were elicited by the same stimulus^52,58^.

The ultimate decision of depositing eggs by a female mosquito demands a complex integration of sensory inputs, from finding a breeding site to assessing its quality, behaviors that have been superficially explored. Recent studies on how volatiles can modulate oviposition behavior^11,13,16,59,60^, and on how mosquito females evaluate salt concentration in the breeding site before making an oviposition decision^10^ have highlighted the role of chemosensation during this critical but vulnerable step in the mosquito life cycle. Our study shows that olfactory conspecific communication can assist female mosquitoes in regulating population density at a breeding site. We expect that further understanding of the communal cues regulating the choice of a breeding site may offer opportunities to manipulate mosquito oviposition behavior to aid vector control.

## Methods

### Biosafety and ethics statement

All research procedures performed in this study followed the NIH guidelines and the Florida International University Environmental Health and Safety guidelines. All maintenance and experiments with genetically modified strains of *Ae. aegypti* mosquitoes were performed in Arthropod Containment Level 2 (ACL2) facilities. The biohazard disposal, laboratory protocols and practices, and equipment were reviewed and approved by the Florida International University Institutional Biosafety Committee (IBC-21-002-CR01).

### Mosquito strains

*Aedes aegypti* mosquitoes from the Orlando strain (considered wild-type) and 5 mutant lines generated in the Orlando background in previous studies (*orco*^*5/5*^ and *orco*^*16/16*^ mutants^14^, *Ir8a*^*attP/attp*^ and *Ir8a*^*DsRed/DsRed*^ mutants^15^, and *Gr3*^*cfp/cfp*^ mutants^16^) were used. Crosses between homozygous *orco*^*5/5*^ and *orco*^*16/*16^ or *Ir8a*^*attP/attP*^ and *Ir8a*^*DsRed/ DsRed*^ mutant lines were performed to obtain *orco*^*5/16*^, or *Ir8a*^*attP/DsRed*^ heteroallelic mutants, respectively*. Gr3*^*cfp/cfp*^ mutants were maintained as homozygous lines.

For the confirmation of the mutant genotypes, mosquito genomic DNA from *orco*^*5/5*^, *orco*^*16/16*^, *orco*^*5/16*^*, Ir8a*^*attP/attp*^, *Ir8a*^*DsRed/DsRed*^, and *Ir8a*^*attP/DsRed*^ mutants, and Orlando wild-type (control) was purified using DNeasy Blood & Tissue kit (Catalog #69506, QIAGEN). PCR amplification was done using GoTaq® Green Master Mix (#M7123, Promega) in 25 uL reactions containing 1 uL of 10 uM of each primer. The primer pairs for *orco* locus were ORCO_TIDE_F2_T7:

5’- TAATACGACTCACTATAGGGGAACGTCCAACCGACAAAAT-3’ and ORCO_TIDE_R: 5’- CGACGACGGATAGCACTGTA-3’, and *Ir8a* locus IR8a_TIDE_F2: 5’-TAATACGACTCACTATAGGGTGGTCGGTTTGATCTTCTGAC-3’ and IR8a_TIDE_R2: 5’-ACGTGGTCCACATCTTTGACT-3’. The thermocycler program for *orco* locus amplification was 95 °C for 3 min, followed by 35 cycles of 95 °C for 30 s, 55 °C for 30 s, and 72 °C for 45 s, and a final extension of 72 °C for 5 min. The program for *Ir8a* locus was 95 °C for 2 min, followed by 33 cycles of 95 °C for 20 s, 54 °C for 10 s, 72 °C for 4 min, and a final extension of 70 °C for 1 min. The amplified products were cleaned using Monarch® PCR & DNA Cleanup Kit (New England Biolabs) and Sanger sequenced for subsequent TIDE analysis^61^. For *Gr3*^*cfp/cfp*^ genotype confirmation, the larvae for all the batches from *Gr3*^*cfp/cfp*^ homozygous mutants were screened and confirmed for cyan fluorescence^40^.

### Insect rearing

Wild-type and mutant mosquitoes were maintained in the insectary room at 27 ± 1 °C with 70 ± 10% relative humidity in 14 h light:10 h dark regime with lights on at 8 am. Eggs were hatched at room temperature in a vacuum-sealed Mason jar containing 0.5 L of pre-boiled deoxygenated deionized (DI) water with a dissolved tablet of fish food TetraMin tropical tablets (Tetra, Melle, Germany). Hatched L1–L2 larvae were sorted to a density of 200 per rearing pan (5.3 L polycarbonate food pan, Carlisle, Oklahoma, USA) containing 2 L of deionized water and fed TetraMin tablets until pupation. The pupae were transferred to ceramic ramekins containing deionized water and contained in a 30 × 30 × 30 cm BugDorm-1 insect rearing cage (MegaView Science Co., Ltd., Taiwan). Emerged adults were kept in a 1:1 male:female ratio and fed with 10% sucrose solution *ad libitum*. To generate eggs for colony maintenance and experiments, 5- to 10-day-old adult female mosquitoes were artificially blood-fed using a 50 mm glass feeder (# 1588-50, NDS Technologies, Vineland, NJ) covered with stretched Parafilm™ M wrapping film (Bemis™, Thermo Fisher Scientific™, Waltham, MA) pre-rubbed on human skin to enhance mosquito attraction. The feeder was filled with 3 mL of pre-warmed (37 °C) defibrinated whole sheep blood (#R54020, Remel Inc, Thermo Fisher Scientific™, Lenexa, KS) supplemented with 0.4 mM ATP (#34369-07-8, ACROS Organics™, Thermo Fisher Scientific, Pittsburgh, PA). The feeder was coupled to a 37 °C heated circulating water bath and females were allowed to access the feeder through the mesh panel of the cage for 1 h. A white porcelain ramekin lined with Whatman #1 qualitative filter paper (#1001-055, GE Healthcare, Buckinghamshire, UK) and half-filled with deionized water was placed into the cage 3 days after the blood meal for the females to lay eggs.

### Behavior experiments

All the behavioral experiments were performed in a behavior room maintained at 25 ± 1 °C with a lower relative humidity of 50 ± 5% to enhance water detection by the female mosquitoes. Experiments were performed in constant lights-on conditions for 24 h for the two- or four-choice water trap assays or for 48 h for the oviseekmeter assays (room illumination ranging from 700 to 1500 lumens, measured with a Holdpeak Aoputtriver AP-881D digital lux meter, Zhuhai Holdpeak instrument Co., Ltd., Guangdong, China). In two-choice water trap assay trials with low light conditions (0.02 to 0.05 lumens), lights were off for 24 h. For all the assays, mortality was recorded due to the long duration of the experiments (Supplementary Figs. 1c, h, 2b, f, 3b, f, 4b, d, 5b, 6b, e). All behavior experiments were performed with gravid females (breeding site seeking females) assayed 3 days after the blood meal (72–82 h). Sugar-fed females were used only as pre-placed individuals in a chamber of the oviseekmeter, or to validate the oviseekmeter assay (sugar-fed, Supplementary Fig. 3c). To obtain gravid females, 5- to 10-day-old females were deprived of 10% sugar solution for 16–20 h but allowed unlimited access to water during this period. Sugar-starved females were blood-fed as described above, and fully engorged females were visually inspected and sorted by aspiration using a mouth aspirator with HEPA filter (model 612, John Hock Company, Gainesville, FL). The confirmed blood-fed females were transferred to a new rearing cage (BugDorm-1) containing 10% sucrose for *ad libitum* feeding, and the cage was kept in the insectary for 3 days until the behavior assays were performed. All the assayed mosquitoes were sacrificed after the behavior experiments.

### Water trap assays

To characterize and quantify the aggregation levels of the breeding site-seeking gravid females or eggs laid in the trials, a modified water trap assay^21^ was used in two- and four-choice configurations inside the BugDorm-1 cage. Each trap placed at opposite corners (two-choice) or at each corner (four-choice) consisted of a rectangular prism of 1296 cm^3^ (9 cm length × 9 cm width × 16 cm height) built with 4 transparent or opaque (black) acrylic plates (16 cm length × 9 cm width, and 0.5 cm thick). Each trap had a removable 9 cm×9 cm square acrylic lid, with a 6 cm diameter center hole and a square inner fitting of 7.8 cm × 7.8 cm (0.5 cm thickness) to hold the lid against the prism. A 5 cm long funnel with 6 cm diameter at the top hole and 2 cm diameter at the bottom hole was made with a flexible polypropylene black screen (1 mm mesh hole) and was applied against the inner wall surface (1 cm) of the lid hole. The meshed funnel extended 4 cm from the lid into the trap and was used to impede females from escaping the trap once a choice was made.

For the breeding site, a 30 mL ceramic ramekin (3 cm height, 5 cm top, and 4.5 cm bottom diameters—1 oz HIC Mini Butter Crock Ramekin, HIC Harold Import Co., Lakewood, NJ) was lined with 3 half-circle-shaped filter papers (Whatman #1) and filled with 10 mL of deionized water. Depending on the assay, traps housed uncovered or mesh-covered ramekins. Mesh-covered ramekins were produced by covering the open side of the ramekin with 2 squared layers (14 cm × 14 cm) of polyester white tulle fabric (Craft & Party). Both layers were stretched on the top before being secured to the ramekin with a rubber band. The ramekin was filled with 10 mL of deionized water by using a motorized pipette and a sterile serological pipet. Mesh-covered ramekins had the mesh dried with a Kimwipe tissue (KimTech Science, Kimberly Clark Professional, Roswell, GA) before the assay to remove any potential moisture. The mesh layers covering the ramekins blocked the females from contacting the water and prevented oviposition. In assays without trap, uncovered ramekins were used so the aggregation could be quantified by the number of eggs laid. All procedures to prepare traps and ramekins were done with gloves. Prior to use, all assay components were washed with odorless liquid dish detergent (Seventh Generation, Inc., Burlington, VT), rinsed in warm water, and finally reinsed in deionized water before being allowed to dry.

### Two or four-choice trials

For the two-choice water trap assay, 35 gravid females contained in the BugDorm cage assembled with the standard configuration were acclimated in the behavior room for 30 min. Ramekins were placed into the cage at opposite corners and immediately covered with traps. After 24 h, the number of gravid females inside each trap, out of the traps, and dead ones were recorded.

For the four-choice assay, the BugDorm cage mounted with 4 pieces of lateral mesh panels was placed in the behavior room without the top cover panel in place. Four transparent traps, each housing a 10 mL-filled ramekin, were placed at the corners of the cage. Gravid females were cold anesthetized at 4 °C, and 35 females were sorted into a 100 mm glass petri dish and placed on ice. After 15 min, the plate with the cold-anesthetized females was placed at the center of the cage. The top panel of the cage was immediately fitted before the females started to move.

After 24 h, the numbers of non-trapped, dead, and trapped mosquitoes in each trap were counted. Following recording the results in two- or four-choice assays, the cages were immediately transferred to 4 °C with the positions of the choices noted. With the gravid females immobilized, the numbers were recounted for confirmation. In two-choice assays with the opaque traps, traps were lifted after 1 h at 4 °C, and the trapped females were counted. For all the assays, the ramekin position was noted before it was recovered from inside the traps and stored at 4 °C until the eggs were counted (see procedure below). Dead gravid females that were scored inside the traps were counted since a choice was made during the two- or four-choice assays.

### Time-lapsed assays in the two-choice water trap

Time-lapse recordings were used to characterize whether a founder effect can elicit preference for a trap. To allow recordings from the top of the cages, the BugDorm cover panels were modified to obtain transparency for the camera lens. The modification consisted of a centered square-shaped cut of 21 cm × 21 cm on the plastic surface. A 27 cm × 27 cm transparent acrylic square plate was attached to the inner side of the cover by drilling four holes at each corner of the plastic and acrylic pieces. The two pieces were secured together with four bolts. Time-lapse digital cameras (TLC200 Pro, Brinno, Taipei, Taiwan) with 32GB memory cards were used on top of the transparent cover panel to record the two-choice water trap assay. A 3D-printed case was used to stabilize the cameras vertically on the cover. The camera parameters were set to a 1-s capture interval, 30-s frame rate, auto white balance, best image quality, and daylight scene mode. The cold-anesthetized gravid females on a petri dish were placed in the center of the cage. The top cover was returned, and the camera was positioned at the center of the acrylic lid. The recording started before the mosquitoes began to move and lasted for 24 h. The trap positions and the respective numbers of females trapped were recorded. The time-lapse video was watched by an independent blind scorer until the first choice was observed. The PI upon first choice was calculated using the formula PI 1st (Fig. 1h top). The mean PI 1st of the assays was compared against the theoretical value 0 (no preference) to determine whether a significant preference was observed.

### Oviseekmeter components

To develop the oviseekmeter two-choice assay (Fig. 3a), we combined pieces of the BugDorm-1 rearing cage, Pyrex plain 147 mm diameter 60° angle glass funnels, with 75 mm long stems that were 10 mm wide on the inner and 15 mm on the outer diameter (#6120-6, Corning, Glendale, AZ), 710 mL clear PET plastic round deli containers (11.7 cm top diameter × 9.1 cm base diameter × 10.9 cm height - 24 oz, #RD24, Fabri-Kal® AlurTM, Kalamazoo, MI) with clear PET plastic round deli container lids that had an air-tight fitting (inner-fit, #760LRD, Fabri-Kal® AlurTM, Kalamazoo, MI). Before assembly, BugDorm pieces and glass funnels were washed as described above. New plastic containers used as chambers were set up in the device and never reused after an experiment. All pieces were assembled with gloves. The main cage of the oviseekmeter was assembled from 2 mesh panels, 2 front panels, and 2 cover panels from BugDorm-1 cages. The glass funnels were glued to the front panels. Two plastic containers with lids were used as chambers, and the other 2 plastic containers without lids were used as underneath supports for the chambers. Two ramekins were used as oviposition sites. A hot melt glue gun used in the 100 watts settings (#SI-205, Camelot, Montréal, Québec, Canada) and hot glue sticks (Gorilla hot glue sticks, #8401509, The Gorilla Glue Company, Cincinnati, OH) were used to glue the funnels to the front panels as well as the chambers to the funnels and supports as described below.

### Oviseekmeter device assembly

Each front panel with a circular opening of 15.5 cm diameter held a glass funnel with the stem facing the external side of the panel (stockinette sleeve ringside). To attach the funnels to the front panels, they were put apart on a bench with the stems facing up. Both shorter ends of the angled tips were aligned to the bottom of the panels. The panels with the sides of the sleeve ring facing up were fitted on the funnels and hot glued against the panel by completely filling the gaps between both pieces on both sides of the front panels. Each piece was checked to make sure the hot glue sealed the entire rounded area between both pieces. Each choice chamber was composed of 2 plastic containers and 1 inner fitting lid. To build the chamber, 1 container was flipped with the opening facing down. Another container with the opening facing up was hot glued against the bottom part of the container (rounded surfaces aligned), and two sets of chambers were prepared.

To assemble the oviseekmeter, 2 mesh panels were fitted into each other and both pieces were snapped into the bottom panel of the cage. The 2 prepared front panels were fitted into each other and into the mesh panels. The funnel panels were snapped into the bottom cover. Once the 4 lateral pieces were in place, the top cover panel was snapped into the cage. To connect a mounted chamber to the funnel stem, the lateral surface of the top container was aligned to the angled tip of the funnel. The contact area between both pieces was marked on the plastic container. A hole smaller than the funnel stem diameter was drilled on the mark using the pre-heated nozzle of the hot glue gun. The chamber was quickly slid onto the funnel stem when the hole on the container was hot and malleable to generate a tight fitting. The depth of the stem inside the chamber was adjusted to 3 cm. The chamber was sealed to the stem with hot glue and checked for complete sealing.

### Oviseekmeter behavioral assays

All the assays were performed in the behavior room with the conditions described above. The stem of the funnel produced a long path with a narrow entrance to the chambers. Due to this condition, the assays had a duration of 48 h to ensure that the maximum number of gravid females released could pick a choice. For each trial, an oviseekmeter was set up on the day of the trial with new plastic chambers. The other pieces were washed a day before, as described above and dried at room temperature overnight (12–16 h). Every piece was carefully checked for the presence of water before assembly.

Assembled oviseekmeters were placed in the behavior room before the gravid females were prepared for the assay to acclimate to the room conditions. The top panels of the cages were removed and set aside. The lids of the chambers were removed, the 30 mL ramekins with the appropriate condition were put in the center of each chamber, and the lids were placed back. All the gravid females prepared for the assay were cold anesthetized at 4 °C and transferred into a 150 mm glass petri dish placed on ice. They were sorted from the glass dish into a polystyrene 60 mm diameter petri dish and rested on ice until the desired number of females for each group was obtained, and the lid of the plate was placed back. For releasing gravid females in the oviseekmeter, each plate containing the desired number of females was placed without the lid into the main cage of the device at the corner of the meshed panels (opposing the panels with the funnels). The top panels of the cage were quickly returned to the device before the gravid females recovered from cold anesthesia.

The gravid females were allowed to pick a choice for 48 h. After this period, the number of alive and dead females remaining inside the main cage (no choice made), and the number of females inside each chamber (choice made) was recorded. Dead gravid females inside any chamber were scored as a choice made. Gravid females in the stem of the funnel were considered inside the main cage (no choice made).

### Chambers with pre-placed females

For all the oviseekmeter assays with pre-placed mosquitoes in the chamber, 3 or 15 females were placed in one of the chambers. Pre-placed gravid females, whether intact or wingless, as well as the sugar-fed females, were reared alongside the females that were released to make a choice in the assay. The pre-placed and released females were prepared for the trials following the methods described above. To remove the wings from the gravid females, we first cold-anesthetized gravid females on a 60 mm diameter disposable plate. We then used a surgical micro scissor with 5 mm blades (SKU #9600, Vannas scissors, Moria, Doylestown, PA) to cut off the wings without damaging the underlying muscles. All the assays had a 30 mL mesh-covered ramekin containing 10 mL of deionized water placed in each of the chambers. To pre-place females, cold-anesthetized mosquitoes were placed onto the meshed surface of the ramekins inside the chambers. Immediately after the chambers were set up with the pre-placed females, the anesthetized gravid females were released inside the main cage of the oviseekmeter.

### Choice dynamics in the oviseekmeter

We used the oviseekmeter to time-lapse assays with 30 gravid females over 48 h, taking advantage of the transparency and length of the funnels’ stems. In our attempts in the two-choice water trap assays, it was unfeasible to score numerous mosquitoes over 24 h, due to the size, design, and black-meshed entrances of the traps. A time-lapse digital camera with the same settings described above was positioned on top of the cover lid of the oviseekmeter, with the lens at an angle that covered the distal end of both funnels’ stems and the entrance of both chambers. The cold-anesthetized females on a petri dish were placed at the opposite corner of the funnels’ rims and the recording started before the females awakened. The recording was stopped after 48 h, and choice results were obtained. The time-lapsed videos were visually scored. A mosquito was considered to have made a choice when it left the distal part of the funnel and entered the chamber. The dynamic of the choices of the gravid females was expressed as the percentage of females trapped in each of the chambers at a time over 48 h, to depict aggregation patterns. The fold change per minute was calculated by dividing the trapping percentages from both chambers in every scored minute in each trial. The mean fold change obtained from all 9 assays was used for plotting the dynamic fold change over 48 h (Fig. 3e bottom). Due to technical limitations related to tracking the flight direction of the mosquitoes in some decisions, the time-lapse video scoring results did not always match the results documented by counting the mosquitoes after the experiment was finished. We then compared the scored and video-scored final fold-change, and no significant difference was found, validating the video-scored counts (Supplementary Fig. 3g).

### Carbon dioxide assays with the oviseekmeter

We modified the oviseekmeter to allow air and carbon dioxide influx into the choice chambers. A hole was drilled in the plastic cup chamber to introduce a plastic straight barbed hose fitting. This piece was attached to the cup with hot glue. The inlet was positioned in front of the funnel stem insertion, 3 cm below the cup rim. To deliver air or air and carbon dioxide mixture to the chambers, we used gas cylinders (Airgas_→_, Radnor, PA) containing compressed air (76.5–80.5% Nitrogen; 19.5–23.5% Oxygen composition, UN1002) and carbon dioxide (100% CO_2_ composition, UN1013) for the assays. A high purity dual stage low flow regulator R3 Series (model R31BMK-DSK-C500-11-D, GENTEC®, Chino, CA, USA) was connected to the compressed air cylinder. A carbon dioxide compressed gas regulator (model FS-100, Fisher Scientific, USA) was connected to the carbon dioxide cylinder. PVC laboratory tubing (R-3603 and S3™ B-44-3, Tygon^®^, Saint-Gobain Corporation, Courbevoie, France) and straight and tee barbed hose adaptors were used for all the connections in the system. The compressed air was humidified by passing through a 1000 mL glass filtering flask containing 400 mL of deionized water and was connected to the main flowmeter (model VFB-69-SSV, Dwyer Instruments, Michigan City, IN, USA) with an airflow of 4.0 L/min. From the main flowmeter, the air tubing was split into two lines. One line was split again into three flow lines, each routed to an independent flowmeter with a flow of 0.2 L/min (model VFA-21-SSV, Dwyer Instruments). Each flow line supplied a control chamber, two control chambers for the oviseekmeter used for the control assays, and one chamber for the oviseekmeter used for the experimental assays. The other air tubing line split was connected to a standard pneumatic manifold serving as a mixer (2 Inlet Ports, 2 Outlet Ports, PCM10-125-02B, Polycoon©, Plymouth, Minnesota, USA). The carbon dioxide tubing line was connected to a flowmeter with a flow of 0.1 L/min (model VFA-21-SSV, Dwyer Instruments) and then to a bubble counter valve (needle valve carbon dioxide regulator for aquarium system) set to 100–120 bubbles/min (model: CW02062-03, EECOO, Mainland China) to further reduce the carbon dioxide flow. The carbon dioxide line was routed to the same manifold mixer (PCM10-125-02B) to mix with the compressed air line. The 2 outlet lines with the air and carbon dioxide mixture were connected to the two inlets of a 6-way manifold (PCM10-125-10B) with all outlets blocked but one, which connected a line to a flowmeter with a flow of 0.2 L/min (model VFA-21-SSV). The outlet line with mixed air and carbon dioxide supplied one chamber of the experimental oviseekmeter used in the assays.

A hand-held carbon dioxide meter (model CO2-100, Amprobe, Everett, WA, USA) was used to monitor carbon dioxide levels in the assay. To measure the levels in the chambers, a square was cut on the cup lid to insert the bottom sensor of the meter. The opening was sealed around with tape, and the lid was returned in place. To measure the carbon dioxide levels at the choice points in the main cage, the carbon dioxide meter sensor was positioned at the entrances of the funnel rims, and the level was measured until stabilization. The control chambers with only air influx had a carbon dioxide concentration of 100–200 parts per million (ppm), and the corresponding funnel entrance in the main chamber had 450–500 ppm. The chamber with air mixed with carbon dioxide had a 1400–1800 ppm of carbon dioxide concentration, and the connected funnel entrance in the main chamber had 600–700 ppm. The room had a background level of carbon dioxide from 450–550 ppm before the experiment started and 500–800 ppm at the end of the assay.

The assays were performed in a behavior room at 25 ± 1 °C with 50 ± 10% relative humidity in constant lights-on conditions for 24 h. The behavior room had the door closed during the trials, but the ventilation system of the room was unobstructed to allow air renovation during the assays. Each chamber of the oviseekmeters had a meshed ramekin with 10 ml of deionized water. The compressed air and carbon dioxide flow were open and stabilized at the conditions described above, and thirty cold-anaesthetized gravid females were placed inside the cage. After 24 h, the number of females in the main cage and in both chambers were recorded, as well as the number of dead females. The experimental chamber with supplemented CO_2_ was randomized throughout the assays, connecting the supply line in a different chamber for each set of assays.

### Egg counting

Each ramekin containing eggs was recovered from the water trap assay and stored in a rearing pan with a lid at 4 °C until analyzed. Eggs on the surface of the water were filtered onto the surface of a 125 mm filter paper (Whatman #113) and rested on a porcelain Buchner funnel (114 mm plate, CoorsTek®, Golden, CO) coupled with a vacuum filtration set. The three semicircular filter papers (Whatman #1) lining the ramekin (Fig. 1b) were recovered and the eggs were rinsed from their surfaces to the filter papers with a water-filled wash bottle. The ramekins were rinsed until the remaining eggs were filtered, and clumped eggs were spread out on the filtering paper with additional water rinses.

The filtering paper containing all the eggs was sandwiched between two squared sheets of polypropylene film (#S-8575, Uline, Pleasant Prairie, WI) and was scanned using a CanoScan LiDE 210 (Canon, Canon USA Inc., Newport News, VA) with color (million), detect enclosing box, and 1000 dpi settings checked. The obtained TIFF images were converted to 8-BIT (black and white) and analyzed by Fiji/ImageJ software (NIH) following a method previously described^62^ with modifications. The SET SCALE function was removed (ANALYZE: SET SCALE submenu), the minimum method of the AUTO THRESHOLD was selected (IMAGE: ADJUST submenu), and all the other boxes were unchecked. The digital particle areas of the eggs were determined with the ANALYZE: ANALYZE PARTICLES submenu with the following settings: SIZE: 0–INFINITY, CIRCULARITY: 0.00–1.00, SHOW: Nothing, DISPLAY RESULTS: On, CLEAR RESULTS: On, SUMMARIZE: On, and all the other boxes unchecked. The obtained values for each digital particle area were summed and the total value was divided by the trimmed mean of all the particle areas using the TRIMMEAN function in Excel software (Microsoft) to obtain the total number of eggs on the filter paper (inside the ramekin).

### Gravid female preference index (GFPI) and egg-laid index (ELI)

The indices were used to estimate whether gravid females showed a preference between two equal or two unequal choices in the assay, with a range value from −1 to 1, where 0 indicates no preference. The gravid female preference index (GFPI, Fig. 1c, formula on top) was defined as the difference between the number of gravid females in choice A and choice B divided by the total number of gravid females in choice A and B summed. The egg-laid index (ELI, Fig. 1c, formula at the bottom) was used to estimate whether gravid females had a preference to lay between oviposition choices, defined as the difference between the number of counted eggs in the choice A and the choice B divided by the total number of counted eggs in the choice A and B summed.

### Statistics and reproducibility

All the binomial simulations and statistical analyses were obtained using Graph Pad Prism version 10.0.2. For the statistical analyses, every dataset was first evaluated for normality with the D’Agostino-Pearson omnibus K2 test, followed by the appropriate parametric or non-parametric tests. The significance level was defined as *p* < 0.05. The statistical analysis details as statistical tests, number of trials (n), and number of insects are indicated in the figure legends.

The obtained Gravid Female Preference Index or Egg-laid preference index was tested against the theoretical value 0 for detection of preference using a two-tailed One-sample *t*-test for parametric data or a Wilcoxon signed-rank test for non-parametric data. The preference difference among groups was tested using Ordinary one-way ANOVA followed by Tukey’s multiple comparison tests when the data were parametric, or Kruskal–Wallis followed by Dunn’s multiple comparison test when the data was non-parametric.

For the distribution of traps or chambers with more than 50% of females trapped or eggs laid, all the values from observed traps or chambers with more than 50% in each trial were used for the comparison against the simulated binomial data. For each simulated dataset, the number of events and replicates were adjusted to match the number of mosquitoes and trials performed in the assays. The random number obtained by the binomial simulation for each replicate was converted into a percentage relative to the total of events. The percentage was used for choice 1, and the percentage of choice 2 was estimated relatively. The highest percentage between the simulated choice 1 and choice 2 from each replicate was used for the comparison with the observed data. The comparisons were performed using pairwise (Unpaired *t*-test) or multiple comparisons against the simulated control by Ordinary one-way ANOVA followed by Dunnet’s multiple comparison test for parametric data or Kruskal-Wallis followed by Dunn’s multiple comparisons test. All data were represented in the figures as mean ± standard error of the mean.

### Supplementary information

Supplemental Figures

Description of Additional Supplementary Files

Supplementary Data 1

## Data Availability

The authors declare that all other data supporting the findings of this study are available within the paper and in Supplementary Data 1. Time-lapse videos are available on the Dataverse repository for the first-choice assays—10.7910/DVN/69COMS^63^, and for the oviseekmeter assays—10.7910/DVN/BZY3YJ^64^.
