# Supplementary material for: Female Aedes aegypti mosquitoes use communal cues to manage population density at breeding sites
Source: Commun Biol. 2024 Jan 31;7:143. doi: 10.1038/s42003-024-05830-5 (PMC10830494; doi:10.1038/s42003-024-05830-5)
Supplement: Supplementary file 1 — Supplemental Figures [file 42003_2024_5830_MOESM1_ESM.pdf]

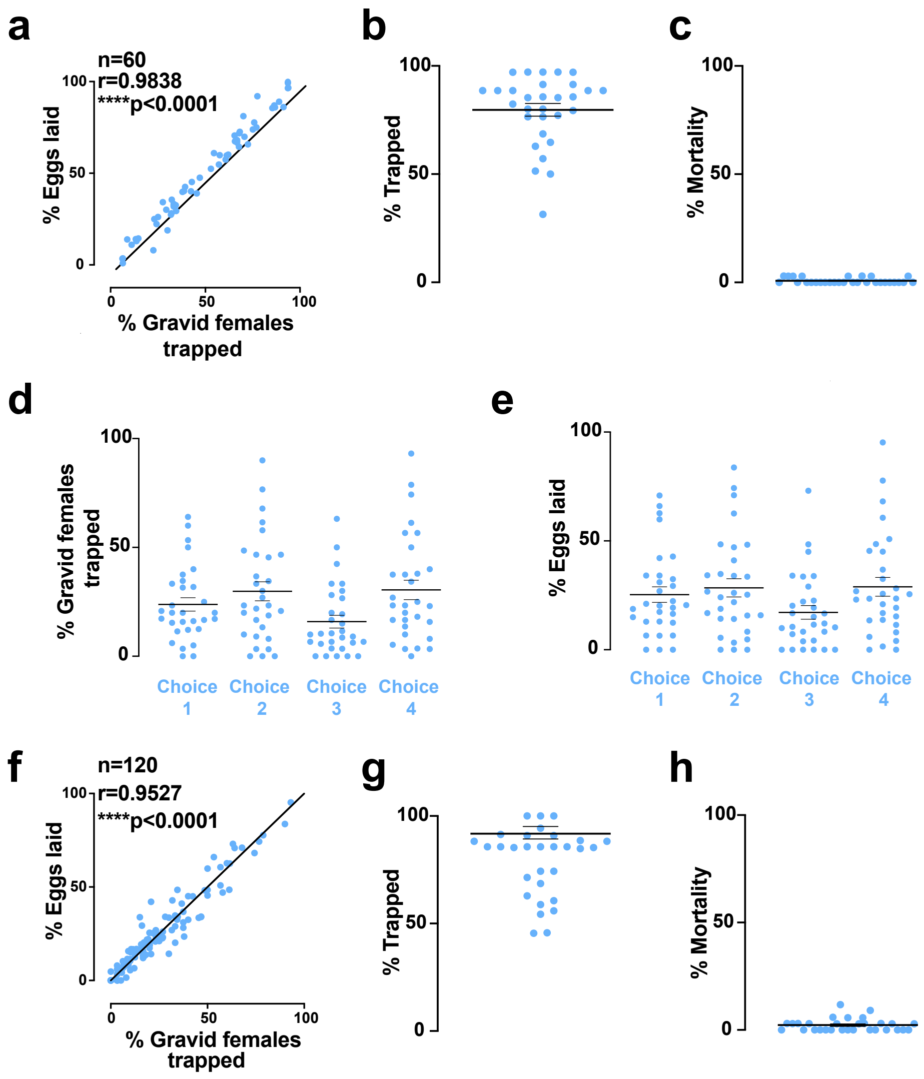

Supplementary Figure 1. Percentages of gravid females trapped and eggs laid are strongly correlated. (a-f) Analyses performed with the results of the two-choice water trap trials shown in Figure 1d (n=30 trials, 35 mosquitoes per trial, 24 hours assay) **(a)** Positive and significant Spearman correlation (r=0.9838, ****p<0.0001) between the percentages of gravid females trapped and eggs laid in each trap over all trials. **(b)** Percentage of females trapped in each trial. **(c)** Percent mortality. **(d-h)** Analyses performed with the results of the four-choice water trap trials shown in Figure 1f (n=30 trials, 35 mosquitoes per trial). No significant differences were observed among Choice 1, Choice 2, Choice 3, and Choice 4 for **(d)** the percentage of females trapped (p=0.1250) or **(e)** eggs laid (p=0.1092) by the Friedman test followed by Dunn's multiple comparison test. **(f)** Positive and significant Spearman correlation (r=0.9527, ****p<0.0001) between the percentages of gravid females trapped and eggs laid in each trap over all trials. **(g)** Percentage of females trapped in each trial. **(h)** Percent mortality. Each blue dot represents one trial, black long horizontal bars denote mean, and black short lines represent standard error.

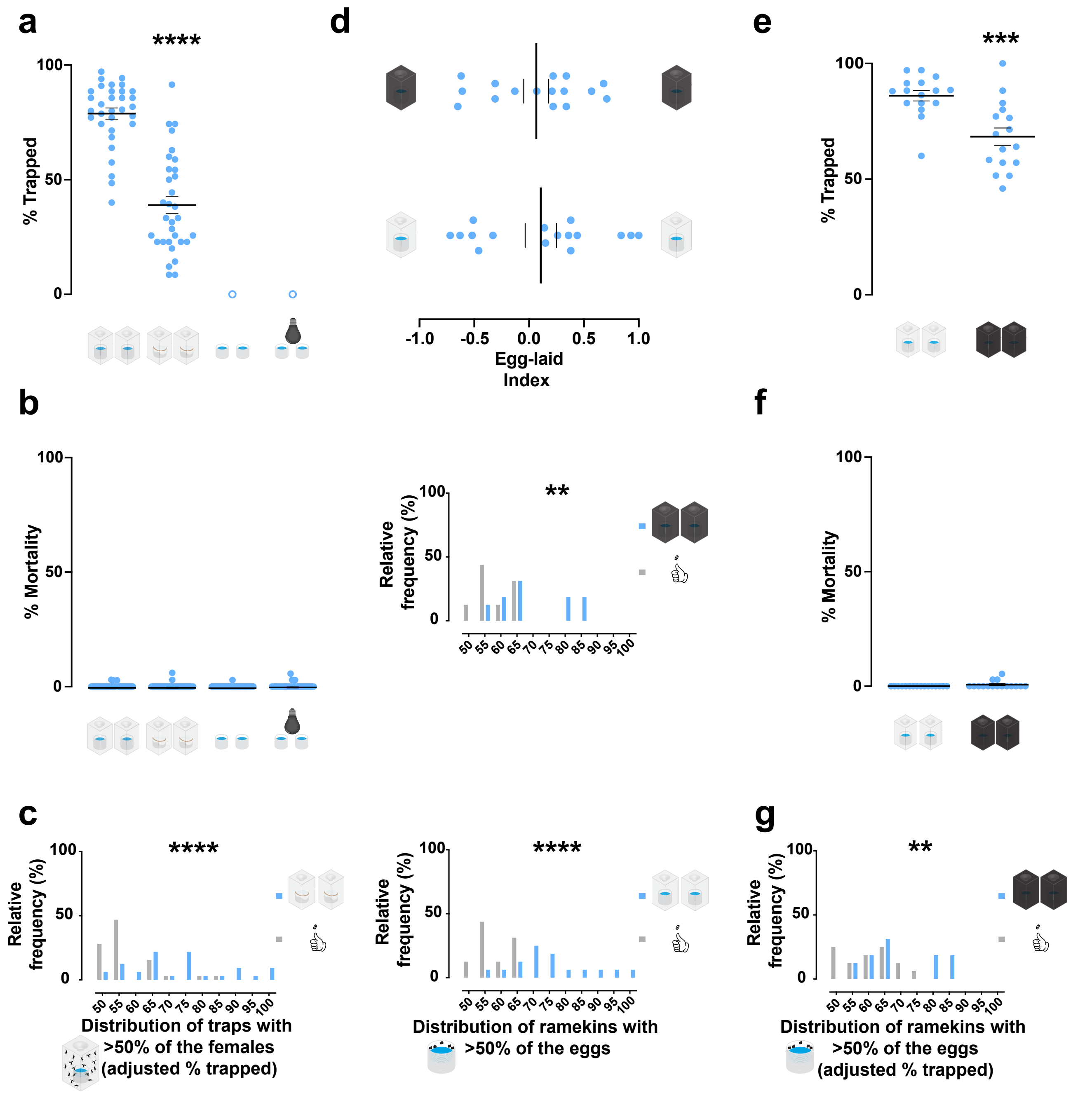

Supplementary Figure 2. Percentage of trapped females reduces when ramekins are occluded or traps are opaque, but aggregation remains. (a and b) Analyses performed with the results of the two-choice water trap trials shown in Figure 2a (n=32 trials, 35 mosquitoes per trial, 24 hours assay). **(a)** Percentage of trapped females differed significantly between water filled ramekins and mesh-covered water-filled ramekins by Mann-Whitney test (****$p<0.0001$). **(b)** Percent mortalities are not different among all the conditions tested by Kruskal-Wallis test ($p=0.5439$). **(c)** Expected (gray bars) or observed (blue bars) distributions of chambers with aggregated gravid females (>50%) in all trials. The observed mean percentage for gravid females was significantly higher than the simulated data adjusted by the mean percentage of trapped females (****$p<0.0001$) by Mann-Whitney test. **(d top)** Egg-laid index from two-choice water trap assay with transparent or opaque trap pairs containing water-filled

ramekins (n=16 trials, 35 females per trial, 24 hours assay). No significant egg-laying preference to either equal choice for transparent (p=0.4650) or opaque (p=0.5716) traps was detected against the theoretical value (0) by One-sample t-test. **(d bottom)** Expected (gray bars) and observed (blue bars) distributions of ramekins with aggregated eggs (>50%) in all trials. The observed mean percentage was significantly higher than the simulated data for transparent (****p<0.0001) or opaque (**p=0.0045) by Ordinary one-way ANOVA followed by Dunnet's multiple comparisons test. **(e and f)** Analyses performed with the results of the two-choice water trap trials shown in Supplementary Figure 2d (n=16 trials, 35 mosquitoes per trial, 24 hours assay) **(e)** Percentages of gravid females trapped in assays with opaque traps are significantly lower than assays with transparent traps by Mann Whitney test (***p=0.0004). **(f)** Percent mortalities between assays with transparent and opaque traps is not significant by Mann Whitney test (p=0.2258). **(g)** Expected (gray bars) or observed (blue bars) distributions of chambers with aggregated gravid females (>50%) in all trials. The observed mean percentage for gravid females was significantly higher than the simulated data adjusted by the mean percentage of trapped females (**p<0.0063) by Unpaired t-test. Each blue dot represents one trial, a gray dot represents one simulated trial, black long horizontal or vertical bars denote mean, short horizontal or vertical black lines represent standard error.

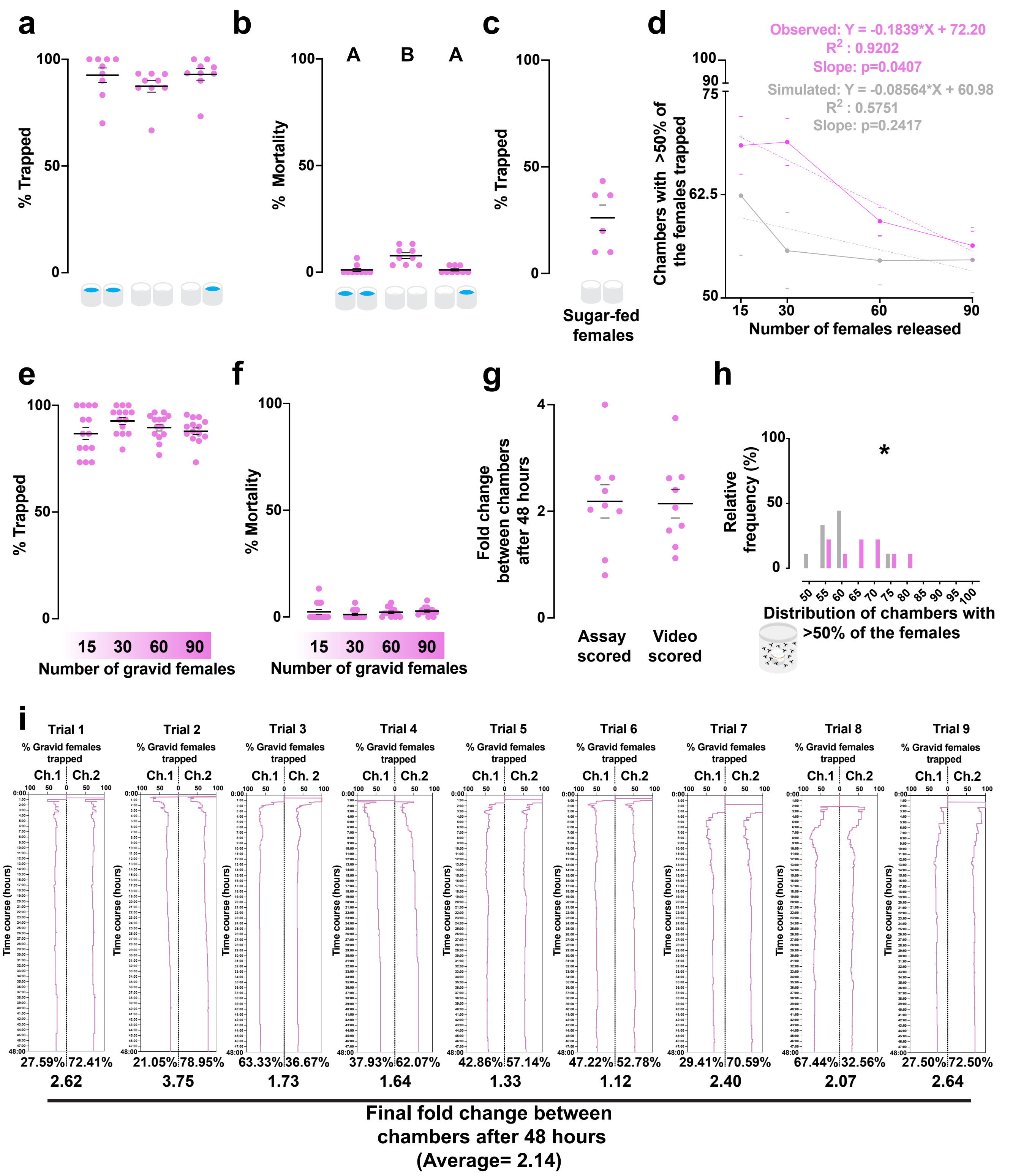

Supplementary Figure 3. Gravid females trapped and mortality were similar regardless of the mosquito density, and the oscillatory choices per trial in time-lapsed assays. **(a-b)** Analyses performed with the results for the oviseekmeter trials shown in Figure 3a (n=9 trials, 30 mosquitoes per trial, 48 hours assay). **(a)** No significant difference in the percentages of gravid females trapped were observed among the conditions (water-filled ramekins in both chambers, empty ramekins in both chambers, or water-filled ramekin in one chamber) by Kruskal-Wallis test (p=0.0581)**. (b)** Percent

mortality of experiments with empty ramekins was significantly higher than water-filled ramekins in both chambers (p=0.0012) or water-filled ramekin in one chamber (p=0.0015), but not between water-filled ramekins in both chambers and water-filled ramekin in one chamber (p>0.9999) by Kruskal-Wallis test followed by Dunn's multiple comparisons test. **(c)** Percentage of sugar-fed females trapped in the oviseekmeter with empty ramekins **(d-f)** Analyses performed with the results of the oviseekmeter trials shown in the Figure 3c (n=14 trials, 48 hours assay). **(d)** Simple regression analysis showing an association (p=0.0407) between the number of females released and chambers with aggregated females (pink line), but not for the simulated data (p=0.2417, gray line). Regression lines for the observed (dashed pink) or simulated (dashed gray) data are indicated. **(e)** No significant differences were observed among the percentages of trapped females when 15, 30, 60, and 90 females were released by Kruskal-Wallis test (p=0.2100). **(f)** No significant differences in the percent mortalities among 15, 30, 60, and 90 released females were observed by Kruskal-Wallis test (p=0.0834). **(g-i)** Results of the time-lapsed trials (n=9 trials, 30 mosquitoes per trial) with the choices scored over 48 hours. **(g)** Levels of fold change obtained by counting the mosquitoes when the assay was completed (assay scored) and by scoring the time-lapse videos (video scored) were not significantly different (p=0.9238) by Unpaired t-test. **(h)** Expected (gray bars) or observed (pink bars) distributions of chambers with aggregated gravid females (>50%) in all trials. The observed mean percentage for gravid females was significantly higher than the simulated data (*p=0.0363) by Unpaired t-test. **(i)** Time-lapsed trials with the pink lines indicate the percentage of mosquitoes trapped in each chamber at a determined time. Final percentages of choices in each trap, fold difference between both chambers, and mean are indicated at the bottom. Each pink dot represents one trial, black long horizontal bars denote mean, and black horizontal short lines represent standard error. Different letters mark whether a group of trials is significantly different.

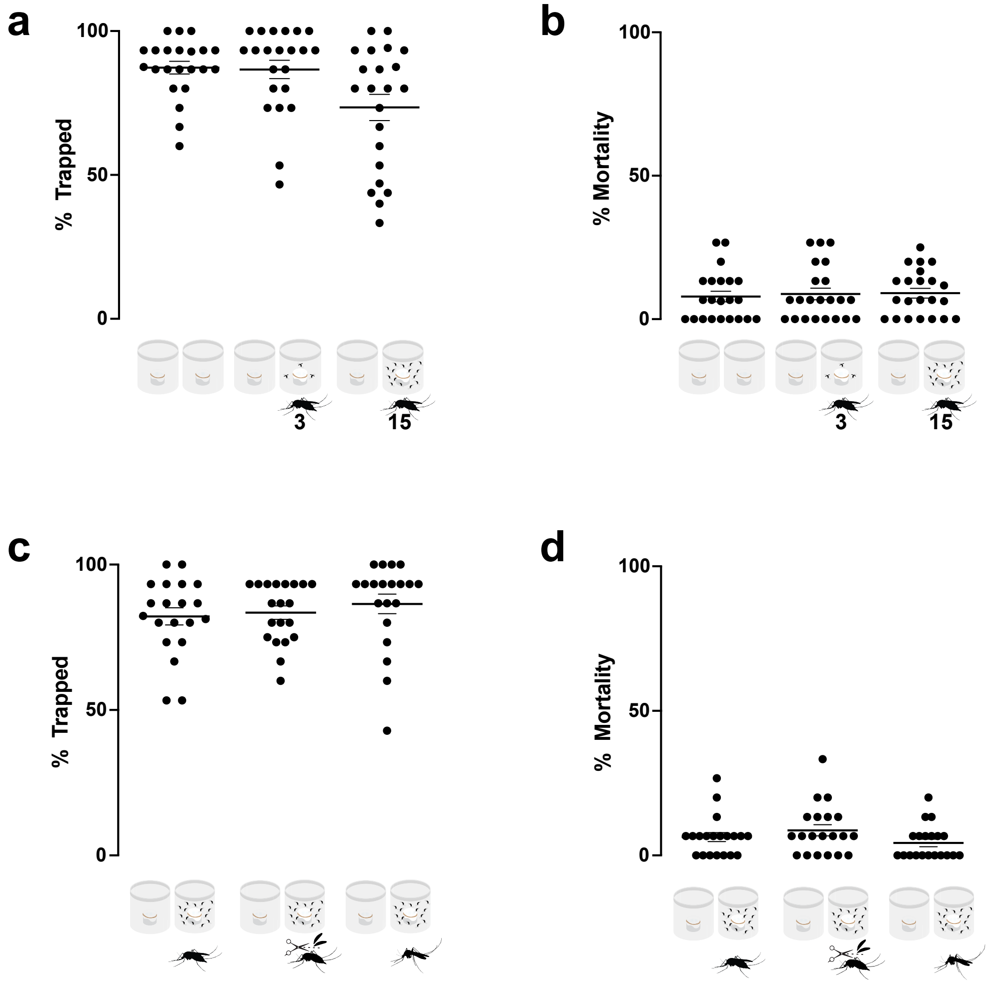

Supplementary Figure 4. Percentages of gravid females trapped and mortality were similar regardless of density or condition of the pre-placed females. (a-b) Analyses performed with the results of the oviseekmeter trials shown in Figure 4a (n=22 trials, 15 mosquitoes per trial, 48 hours assay). **(a)** Percentage of gravid females trapped was not significantly different regardless of having pre-placed gravid females or not (0 versus 3 pre-placed, p>0.9999), (0 versus 15 pre-placed, p=0.1721), and (3 pre-placed versus 15 pre-placed, p=0.0588) by Dunn's multiple comparisons test, after Kruskal-Wallis test detected a significant difference (*p=0.0459) **(b)** Percent mortality is similar regardless of having pre-placed gravid females or not by Ordinary one-way ANOVA (p=0.8931) **(c-d)** Analyses performed with the results of the oviseekmeter trials shown in Figure 4c (n=20 trials, 15 mosquitoes per trial). **(c)** No significant differences were observed in the percentages of gravid females trapped among oviseekmeters with pre-placed intact gravid females, wingless gravid females, and intact sugar-fed females in one of the chambers (p=0.2269) by Kruskal-Wallis test. **(d)** No significant difference in percent mortalities among groups with pre-placed gravid females, wingless gravid females, and intact sugar-fed females in one of the chambers was observed (p=0.1785) by Kruskal-Wallis test. Each black dot represents one trial, long horizontal bars denote mean, and black horizontal short lines represent standard error.

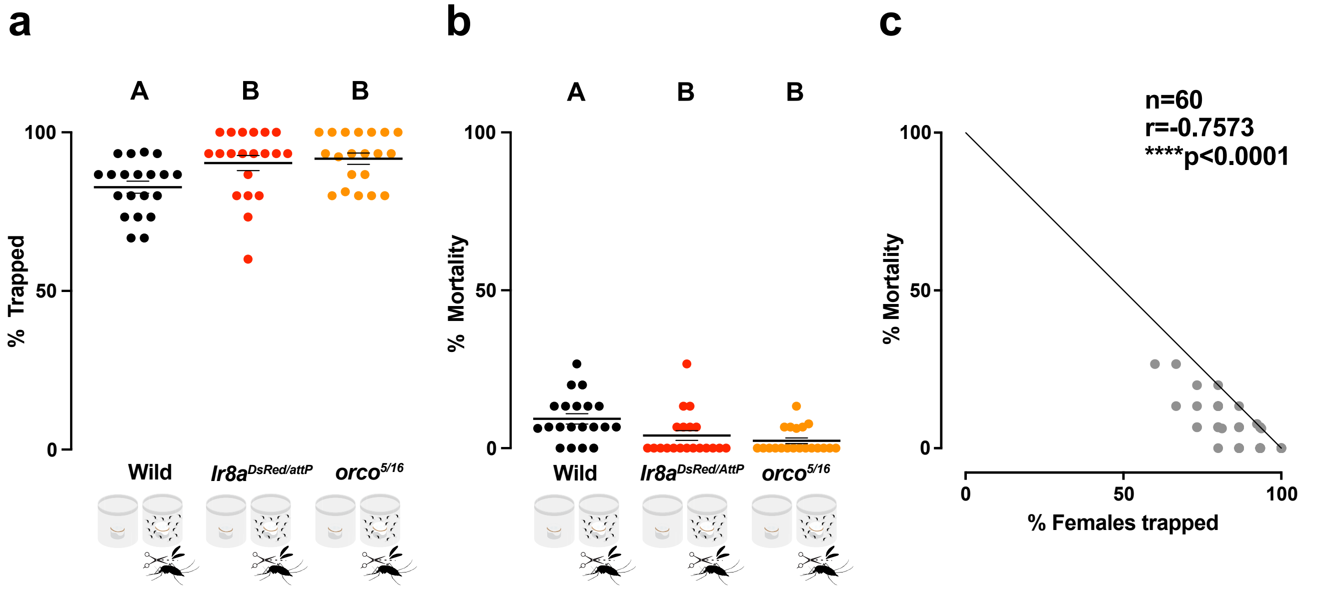

Supplementary Figure 5. ***Ir8a***$^{attP/DsRed}$ **and** ***orco***$^{5/16}$ **mutant gravid females showed higher trapping percentages and lower mortality than wild-type females. (a-c)** Analyses performed with the results of the oviseekmeter trials shown in Figure 5a (n=20 trials, 15 mosquitoes per trial, 48 hours assay). **(a)** Percentages of *Ir8a*$^{attP/DsRed}$ (red dots, p=0.0195) and *orco*$^{5/16}$ (orange dots, p=0.0115) mutant gravid females trapped are significantly higher than the wild-type ones (black dots). Mutant genotypes did not differ from each other (p>0.9999) by Kruskal-Wallis test followed by Dunn's multiple comparison test. **(b)** Percent mortality of *Ir8a*$^{attP/DsRed}$ (red dots, p=0.0164) and *orco*$^{5/16}$ (orange dots, p=0.0026) mutant gravid females are significantly lower than the wild-type (black dots) gravid females. Mutant genotypes did not differ from each other (p>0.9999) by Kruskal-Wallis test followed by Dunn's multiple comparison test. **(c)** Negative and significant Spearman correlation between the percentages of gravid females trapped and percent mortality trap over all trials (r=-0.7573, ****p<0.0001). Each dot represents one trial, long horizontal bars denote mean, and black horizontal short lines represent standard error.

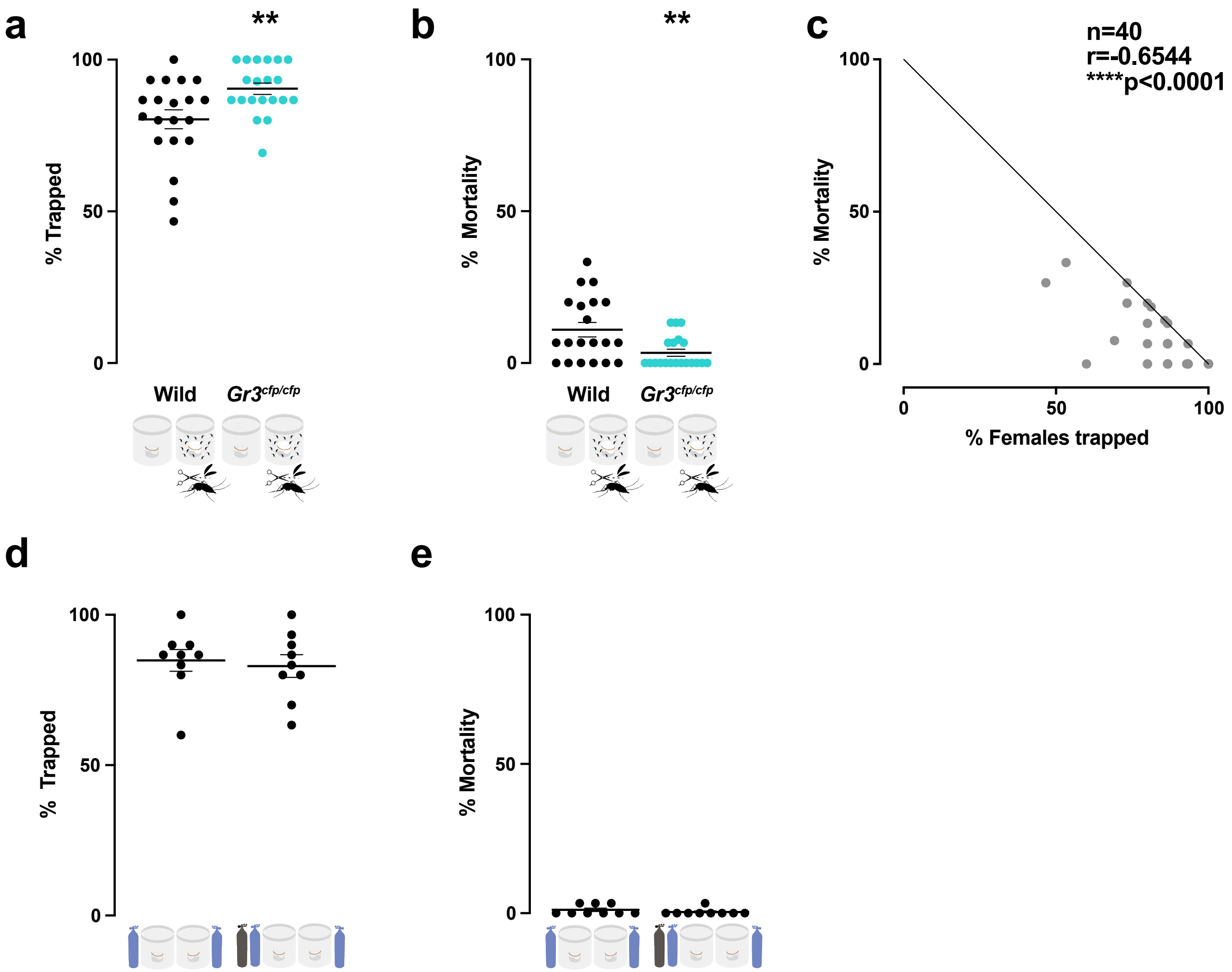

Supplementary Figure 6. *Gr3$^{cfp/cfp}$* mutant gravid females showed higher trapping percentages and lower mortality than wild-type females, and supplemented carbon dioxide does not affect wild-type gravid females. (a-b) Analyses performed with the results of the oviseekmeter trials shown in Figure 6a (n=20 trials, 15 mosquitoes per trial, 24 hours assay). **(a)** Percentage of *Gr3$^{cfp/cfp}$* gravid females trapped is significantly higher than the wild-type gravid females by Unpaired t-test (**p=0.0085). **(b)** Percent mortality of *Gr3$^{cfp/cfp}$* gravid females is significantly lower than wild-type ones by Unpaired t-test (**p=0.0064). **(c)** Negative and significant Spearman correlation between the percentages of gravid females trapped and percent mortality trap over all trials (r=-0.6544, ****p<0.0001). **(d-e)** Analyses performed with the results of the oviseekmeter trials shown in Figure 6c (n=9 trials, 30 mosquitoes per trial). **(d)** Percentage of wild-type gravid females trapped did not differ significantly between trials without and with supplemented carbon dioxide for one chamber only (p=0.6848) by Mann Whitney test. **(e)** Percent mortality of wild-type gravid females is not significantly different between trials without and with supplemented carbon dioxide for one chamber only (p=0.5765) by Mann Whitney test. Each dot represents one trial, long horizontal bars denote mean, and black horizontal short lines represent standard error.
